# Synthesis, application and modelling of spherical magnetic silicon poly-*N*,*N*′-methylenebisacrylamide nanocomposite for effective copper removal from water

**DOI:** 10.1038/s41598-025-26677-8

**Published:** 2025-11-27

**Authors:** Marwa A. Moharram, Mohamed A. Salem, Murat Yılmaz, Mohamed A. Hassaan, Mohamed A. El-Nemr, Ahmed El Nemr

**Affiliations:** 1 Chemistry Department, Faculty of Science, Tanta University, Tanta, 31527 Egypt; 2 Department of Chemistry and Chemical Processing Technologies, Bahçe Vocational School, Osmaniye Korkut Ata University, Osmaniye, 80000 Turkey; 3 National Institute of Oceanography and Fisheries (NIOF), Kayet Bey, Elanfoushy, Alexandria, Egypt; 4 Department of Chemical Engineering, Faculty of Engineering, Minia University, Minia, 61519 Egypt; 5 The Higher Canal Institute of Engineering and Technology, Al Salam 1 - Abu Bakr Al Siddiq Street, Suez, Egypt

**Keywords:** Adsorption, Magnetite, Polymer, Nanocomposite, Copper removal, Water treatment, RSM optimization, Environmental chemistry, Chemical engineering

## Abstract

In this work, a new spherical magnetic silicon-substituted poly (*N*,*N*’-methylenebisacrylamide) (NSM) nanocomposite was synthesized, examined by various known instruments and applied as an adsorbent to eliminate copper (Cu^2+^) from its water solution using batch method experiment. The particles size of NSM composite was ranged from around 24.74 to 28.27 nm. The magnetic NSM nanocomposite are mesoporous, with specific surface area of 63.675 m^2^ g^–1^ and an average pore diameter of 7.6239 nm. The adsorption of Cu^2+^ ions was most efficient at a solution pH 5. The removal process using NSM nanocomposite has been studied in various settings, including initial Cu^2+^ ion concentration, initial pH, and temperature. Using an initial Cu^2+^ ions concentration (50 mg L^–1^) and NSM nanocomposite dose (2.0 g L^–1^), the maximum percent clearance of Cu^2+^ ions was 96.47%. The NSM’s maximum adsorption capacity (*Q*_m_) was 30.30 mg g^–1^. Experimental data were discussed using the Langmuir (LIM), Freundlich (FIM), and Tempkin (TIM) isotherm models. The experimental data from NSM aligns effectively with the LIM model. Several error functions, such as Chi-Squared Error (X^2^), Average Percent Error (APE), Root Mean Square (RMS), Sum of Absolute Errors (EABS), Hybrid Error Function (HYBRID), and Marquardt’s Percent Standard Deviation (MPSD), were applied to validate the isotherm model data. Calculations of the error function suggest that the LIM is the most appropriate for characterizing the adsorption process. Kinetic data were analyzed by fitting pseudo-first-order (PFOM), pseudo-second-order (PSOM), intraparticle diffusion (IPDM) and film diffusion (FDM) models. The PSOM rate model exhibited a robust correlation (*R*^2^ > 0.998) and predominantly governed the adsorption rate. The results show that NSM effectively removes the Cu^2+^ ions from water. Utilizing a response surface methodology analysis to optimize the degradation parameters revealed that a maximum degradation percentage of 52.56 ppm of Cu^2+^ solution and 3.79 g of NSM could be achieved.

## Introduction

Water contamination constitutes a significant threat to human health. Contamination of the global surface water has become a serious problem^1^. Heavy metals pose a risk as they can build in the environment due to various human activities, leading to poisoning^2^. There are five primary sources of heavy metals pollution: pesticides, agricultural contaminants, and metal corrosion; the metallurgical industry involved in alloy production and recycling; waste disposal from incinerators and landfills; geochemical processes; and various manufacturing sources including pigments, dyes, and batteries^3^.

Copper (Cu^2+^) was noticed at high levels in street dust^4^. Cu^2+^ is a critical target for removal due to its detrimental effects on the nervous system^5^. Many techniques are used to remove heavy metal ions, such as flotation, reverse osmosis, coagulation, precipitation, electrochemical separation, ion exchange, chemical oxidation, solvent extraction, and adsorption^6–11^.

Adsorption is among the most efficient and straightforward techniques to eliminate contaminants from aqueous solutions, even with very low concentrations^12^. Thanks to their appropriate surface area, mechanical strength, and pore size dispersion, polymers have been utilized. Thanks to their proper surface area, mechanical strength, and pore size dispersion, polymers have been used.

Polymeric adsorbents also could be regenerated easily in mild conditions. Polymer nanocomposites are more favorable than traditional adsorbents because of their efficient performance, acceptable cost, and easy application^13^. Nanomaterials are excellent absorbents because of their superphysical and chemical properties, which increase adsorption efficacy in removing heavy metals and dyes^14^. However, the regeneration and separation of ordinary adsorbents from aqueous solutions is not easy enough and needs centrifugation with high speed or filtration.

Magnetic adsorbents in an aqueous solution can be effortlessly separated using an external magnetic field^1^. Cu^2+^ is a very widespread contaminant in the environment^15^. It is involved in many applications, such as the fabrication of electric cables and heat pipes, petroleum refineries, chemical catalysis, electroplating, coating materials, and wood pulp production^16^. At relatively high concentrations, copper harms human health and the environment^17^.

Tannic acid (TA) and Cu(NO_3_)_2_ were used in a hydrothermal process to create a unique wrinkled carbon-Cu_2_O nanoparticle adsorbent (HWC-Cu_2_O NPs), which was then used to remove Pb^2+^ from aqueous solutions^18^. Using a one-step hydrothermal process, Makarem et al.^19^ created a microspherical nickel-cobalt-layered double hydroxides-reduced graphene oxide composite (NiCo-LDHs-rGO), which they then employed as an adsorbent to remove Pb^2+^ from aqueous solutions^19^. The GO–WO_3_ nanocomposite was created by modifying graphene oxide (GO) using tungsten oxide nanoparticles (WO_3_ NPs), which were created using the hydrothermal technique^20^. Cu^2+^ was extracted from water samples using the synthesized GO–WO_3_ nanocomposite, yielding a maximum removal percentage of 85%^20^. Although copper (Cu) is a necessary element and catalytic agent of many proteins and enzymes, excessive levels of it can impair fertility, produce anemia and iron insufficiency, and interfere with the absorption of iron and zinc^21^. Numerous studies have shown that Polyphenylsulfone membranes are critical for numerous applications^22^.

This study examined the manufacture and application of magnetic silicon-substituted-Poly(*N*,*N*’-methylenebisacrylamide) (NSM) nanocomposite for the efficient removal of copper (Cu^2+^) from aqueous solutions was studied. The study evaluated variables like adsorbent dose, contact time, and initial pH through batch experiments. Furthermore, the adsorption process’s mechanism and kinetics are elucidated through applying adsorption models. This technique seeks to mitigate detrimental impacts by innovatively and sustainably treating industrial effluent. Also, response surface methodology (RSM) and artificial neural network (ANN) optimization of Cu^2+^ removal from water was conducted.

## Materials and methods

### Materials and equipment

Ethanol (C_2_H_5_OH) (99.99%) and ammonia solution (25%) were purchased from International Company for Sup. & Med Industries, Egypt. Tetraethyl ortho-silicate (99%) and ferrous sulfate heptahydrate (98.5%) were purchased from Alpha Chemical, India. Ferric Chloride (98.5%) and Copper sulphate (CuSO_4_) (99%) were purchased from Fisher Scientific, UK. *N*,*N*’-methylenebisacrylamide (98%) was supplied from Merck, Germany; *N*,*N*-dimethylformamide (DMF) (99.8%) from ADVENT Chembion PVT LTD, India; and ammonium persulphate (98%) from Oxford Lab Chem, India.

The pollutant concentration was measured using a UV-visible spectrophotometer (Analytic Jena, model SPEKOL1300) equipped with glass cells with an optical path length of 1 cm. Experimental procedures employed a SANYO microwave oven (Model EM-D975W, Max Input: 1400 W), a JENCO pH meter (Model 6173), a heating magnetic stirrer (VELP, Code F20500010), and a JS shaker (Model JSOS-500). Surface characterization of the adsorbent was conducted using Fourier Transform Infrared (FTIR) spectroscopy with a platinum attenuated total reflection (ATR) accessory (Model V-100, VERTEX70). This method facilitated identifying functional groups on the surface within the wavenumber range of 400–4000 cm^–1^. The adsorbents’ surface morphology and elemental composition were characterized utilizing a Scanning Electron Microscope (SEM, LEO 1450 VP) equipped with an Energy Dispersive X-ray Spectrometer (EDAX).

### Preparation of NSM

#### Preparation of polymer

A solution was prepared by dissolving 5 g of *N*,*N*’-methylenebisacrylamide in 10 mL of *N*,* N*-dimethylformamide. Ammonium persulphate is used as an initiator. During a few minutes at the beginning of the polymerization process, tetraethyl ortho-silicate (5 mL) was added slowly. The polymerization process continued at 70 °C for three hours with well stirring. The precipitated polymer was separated, washed, and dried at 50 °C.

#### Preparation of magnetic nanopolymer

**Fig. 1:**
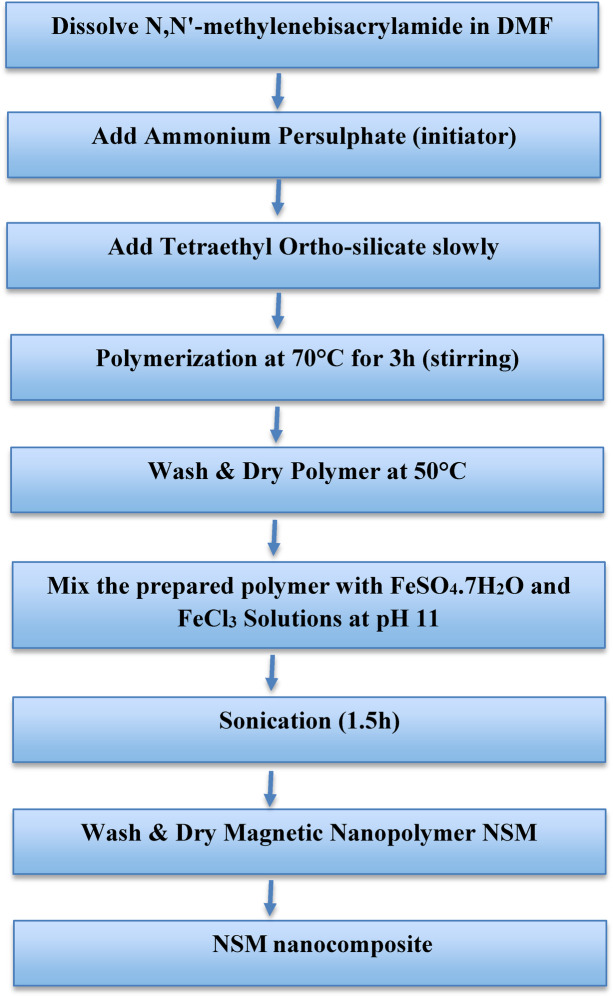
Flow diagram: preparation steps of experimental procedure.

The next step was to add magnetite via the co-precipitation technique. Co-precipitation methods are frequently employed to synthesize magnetic nanoparticles^23^. In a conical flask, the previously prepared polymer was mixed at pH 11 with two solutions of FeSO_4_·7H_2_O (6.75 g dissolved in 100 mL of distilled water) and FeCl_3_ (8.0 g dissolved in 100 mL of distilled water). The mixture was sonicated for 1.5 h, and the resulting magnetic polymer was then isolated, thoroughly washed with distilled water, and dried at 50 °C (Fig. 1).

### Adsorption study

Batch studies quantified copper ion adsorption from an aqueous solution. All adsorption experiments were conducted in shaking instruments at ambient temperatures. The pH values of solutions were adjusted using 0.1 M HCl or NaOH solutions. Various defined starting concentration solutions were added into flasks containing a known quantity of the NSM at ambient temperature (25 °C). The subsequent characteristics were examined to ascertain their impact on Cu^2+^ ions adsorption on the NSM surface: an adsorbent dosage of 2, 2.5, 3.0, 4.0, 5.0, and 6.0 g L^–1^, contact time for 0 to 30 min, pH from 1 to 5, and feed solution concentration of 50, 75, 100 and 150 mg L^− 1^. The pH investigation ended at pH 5 because pH values higher than this cause Cu^2+^ ions to wholly or partially precipitate as Cu(OH)_2_. The initial and equilibrium concentrations were determined using a UV-Vis spectrophotometer at a wavelength of *λ*_max_ 460 nm for Cu^2+^ ions. The suspension was agitated at 200 rpm. At specified intervals, 1.0 mL of the clear solution was extracted and examined using UV-Vis absorption spectroscopy. The reported values represent the average of three replicated experiments. Cu^2+^ ions removal percentage (%*R*) was calculated according to Eq. (1).

1 $$\:\%R=\frac{{C}_{i}-{C}_{e}}{{C}_{i}}\times\:100$$

*C*_*i*_ and *C*_*e*_ represent the Cu^2+^ concentrations (mg L^–1^) corresponding to initial and equilibrium adsorption states. The adsorption capacity of Cu^2+^ ions at time *t* (min) on the NSM adsorbent (*Q*_t_) was determined using Eq. (2):

2 $$\:{Q}_{t}=\frac{\left({C}_{i}-{C}_{t}\right)}{W}\times\:V$$

*C*_t_ (mg L^–1^) represents the concentration of Cu^2+^ ions after time *t*, *V* (L) is the volume of initial feed solution taken, and *W* (g) denotes the weight of NSM used as an adsorbent. The equilibrium adsorption capacity *q*_*e*_ (mg g^− 1^) for the synthesized NSM was determined using Eq. (3):

3 $$\:{Q}_{e}=\frac{({C}_{0}-{C}_{e})\times\:V}{W}$$

*C*_0_ (mg L^–1^) and *C*_e_ (mg L^–1^) denote the initial and the equilibrium concentrations of the Cu^2+^ ions, respectively. *W* (g) indicates the weight of the adsorbent, while *V* (L) represents the volume of the Cu^2+^ solution.

### RSM

Response surface methodology (RSM) was used to study the optimization of factors affecting Cu^2+^ removal in the presence of NSM nanocomposite adsorbent. The Box-Behnken design (BBD) was used, and Design-Expert version 13.0.5.0 was the software used. The starting Cu^2+^ concentration, adsorbent dose, and reaction duration were chosen^24–31^.

Table 1 lists the parameters under study along with their corresponding levels. Cu^2+^ removal percentage (%) was the answer under investigation. Seventeen tests were carried out using various combinations of factors.

**Table 1:** The range of studied parameters used in the optimization process.

Factor	Name	Units	Minimum	Maximum	Mean	Std. Dev.
A	Dose	g/L	2	6	4.00	1.41
B	Time	Min	1.00	30	15.50	10.25
C	Cu^2+^ Conc.	mg/L	50.00	150.00	100.00	35.36

### ANN modelling

The ANN modelling software predicts complex patterns between inputs and outputs by simulating the biological human brain networks and nervous system mechanisms. The ANN type utilized in this study is the feed-forward back-propagation NN (BPNN). The BPNN is used thanks to its flexibility. The input layer (IL), hidden layers (HNs), and output layer (OL) are united to form the ideal BPNN. The inputs and outputs are defined as independent and dependent variables, respectively. The Cu^+ 2^ adsorption ANN approach is simulated and predicted by MATLAB R2015b version. The Cu^+ 2^ adsorption ANN approach training algorithm is the Levenberg Marquart (LM) algorithm with (70%), (15%), and (15%) for training, validation, and testing, respectively. The optimal BPNN had one hidden layer (HL) with 6 hidden neurons. Thanks to its highest *R*^2^ and lowest MSE after testing 4–10 neurons in the training stage. The 3 independent variables were the NSM dosage (mg), time (min), and initial concentration of Cu^+ 2^ (mg/L). The Cu^+ 2^ removal was the only dependent variable^32^.

## Results and discussion

### Characterization of NSM

#### Scanning electron microscopy (SEM)

**Fig. 2:**
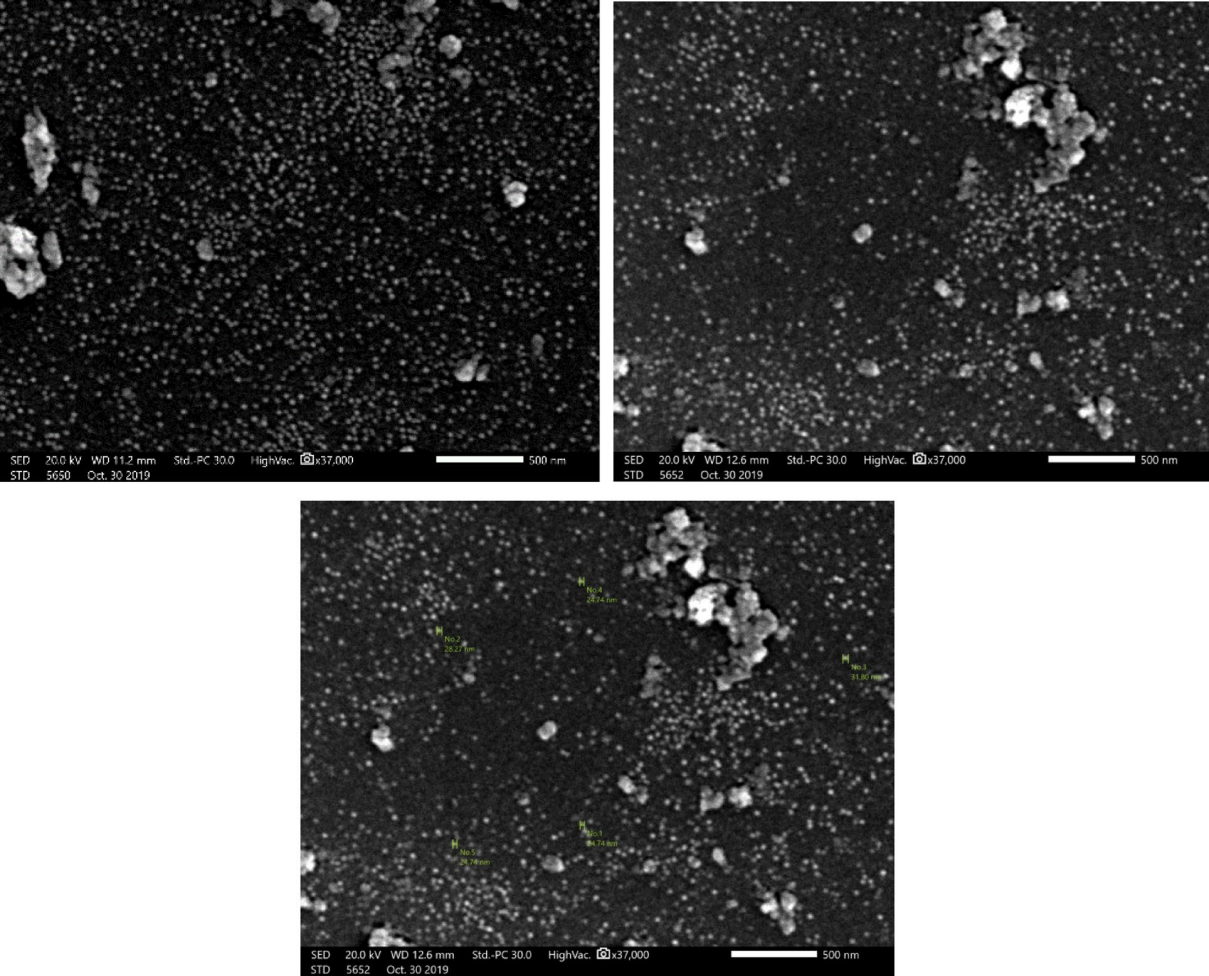
SEM images of NSM nanocomposite adsorbent.

Scanning electron microscopy (SEM) was used to fully characterize the synthesized spherical magnetic silicon poly-*N*,*N*′-methylenebisacrylamide nanocomposite (NSM) morphology. A consistent distribution of distinct, almost monodisperse nanoparticles with a mostly spherical shape was visible in the SEM micrographs (Fig. 2). The particles’ smooth surface and lack of noticeable aggregation suggested that stabilization had been achieved during the synthesis process. The particle size distribution was ascertained by image analysis of the SEM data, which showed that the average diameter of the NSM nanocomposite particles ranged from around 24.74 to 28.27 nm. This small size indicates a high level of control over the synthesis conditions, which is essential to guarantee uniformity and repeatability in the physicochemical characteristics of the material. The discovered nanostructure lends credence to the prospective use of NSM in applications, including magnetic separation, targeted drug delivery, and catalysis that call for nanoscale homogeneity.

#### Thermal gravimetric analysis (TGA) of NSM

**Fig. 3:**
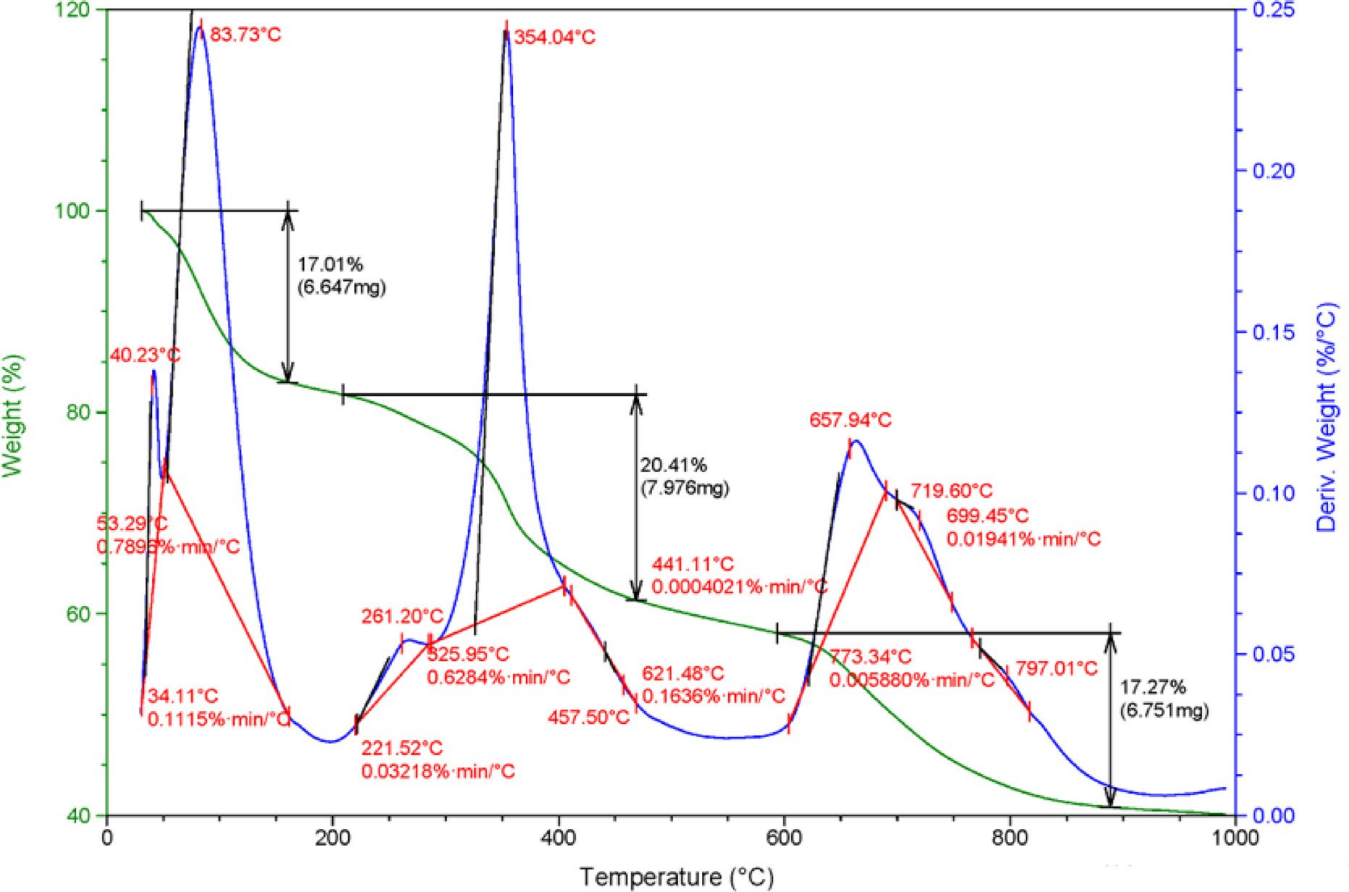
TGA graph of NSM nanocomposite adsorbent.

Thermogravimetric analysis (TGA) is recommended to determine the maximum temperature a material can withstand without decomposition. The NSM heat stability was studied by the TGA curve (Fig. 3). According to the TGA curve, the 1st weight loss of 17.01% is observed below 200 °C, recognized as the removal of physically and chemically absorbed water. Subsequently, 2nd weight loss of 20.41% is observed in the 200–441 °C range, which is recognized as the degradation and depolymerization of polymer units and the imidization of amide groups. During the third mass loss phase, a mass reduction of roughly 17.27% occurs between 600 and 880 °C for NSM with a total weight loss of 60% up to 1000 °C.

#### Fourier transformed infrared (FTIR) of NSM

**Fig. 4:**
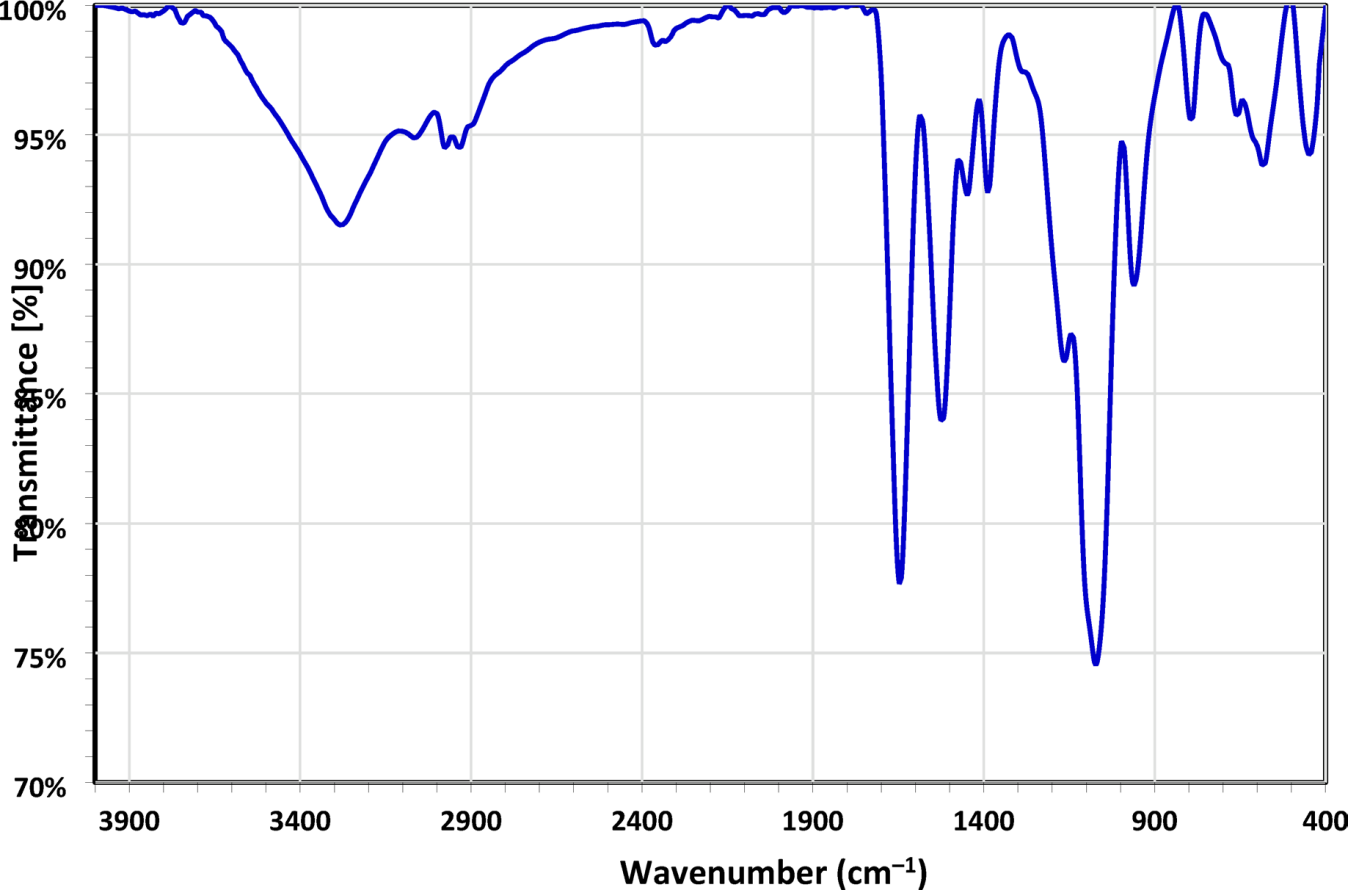
FTIR analysis of NSM nanocomposite adsorbent.

Fourier Transformed Infrared (FTIR) spectroscopy was utilized to characterize the composite, which is considered a reliable technique for identifying specific functional groups^33^. FTIR spectrum of NSM nanocomposite is reported in (Fig. 4). FTIR spectrum exhibits a broad band around 3300 cm^–1^, characteristic of the stretching vibrations of the OH group present on the surface of the magnetic nanoparticles. Bands characteristic of Fe-O stretching vibrations in magnetite are observed in the 400 and 600 cm^–1^^34,35^. Strong bands at 574 cm^-1^ and 420 cm^-1^ in the FTIR spectrum of NSM confirm the presence of magnetite^36^. The carbonyl group (C = O) in the amide is represented in the spectrum by a peak at 1652 cm^-1^^37^ and C = C at 1662 cm^-1^^38^. The peak at 3200 cm^-1^ refers to the N-H stretching of amide, while that at 1616 cm^-1^ signifies the bending vibration of N–H^39^ and C–N at 1446 cm^-1^^33,40^. The broad band at 1086 cm^–1^ corresponds to Si–O asymmetric stretching, while the 498 cm^–1^ peak is attributed to Si–O bending vibrations^41^.

#### BET surface area of NSM

**Fig. 5:**
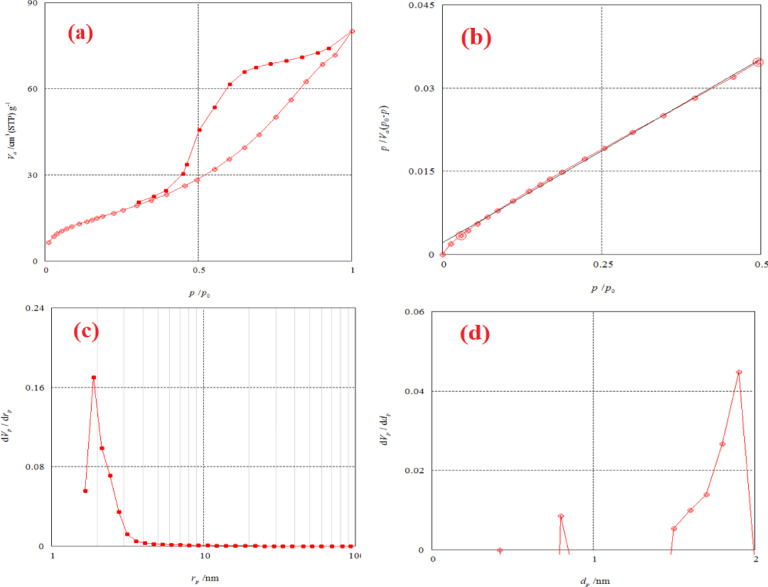
(**a**) Adsorption-desorption isotherm graph, (**b**) BET surface area analysis graph, (**c**) BJH pore size distribution analysis graph, and (**d**) MP analysis graph of NSM nanocomposite adsorbent.

The effectiveness of materials such as catalysts, membranes, and sorbents is primarily determined by their physical properties, particularly porosity and surface area (Table 2). The physical structure of nanomaterials can be characterized using various applications of gas adsorption data, one of which is the Brunauer-Emmett-Teller (BET) theory. The surface area of NSM adsorbent was analyzed using the BET method, derived from nitrogen adsorption-desorption isotherms^42^. Figure 5 shows a hysteresis loop characteristic of a type IV isotherm, indicating that the pores in the magnetic NSM nanocomposite are mesoporous, with an average pore diameter of 7.6239 nm^43,44^. The adsorption isotherm is typically represented graphically, with the amount adsorbed graphed versus the equilibrium relative pressure (*p*/*p*°)^44^.

**Table 2:** BET, BJH and MP analysis of NSM nanocomposite adsorbent.

Type of analysis	Parameter	Data value	Unit
BET	Monolayer volume (V_m_)	14.63	cm^3^(STP)g^–1^
	Energy constant (the first layer) (C)	32.112	-
	Mean pore diameter (MPD)	7.6239	nm
	Total specific surface area (a_s, BET_)	63.675	m^2^ g^–1^
	Total pore volume (TPV)	0.1214	cm^3^ g^–1^
BJH	Mesopore volume (V_p_)	0.1407	cm^3^ g^–1^
	Mesopore area (a_p_)	117.48	m^2^ g^–1^
	Mesopore distribution peak (r_p, peak_)	1.88	nm
MP	Total specific surface area (a_1_)	48.7	m^2^ g^–1^
	External surface area (a_2_)	96.103	m^2^ g^–1^
	Micropore volume (V_p_)	0.00144	cm^3^ g^–1^
	Micropore distribution peak (d_p, peak_)	1.90	nm

#### XRD analysis of NSM

**Fig. 6:**
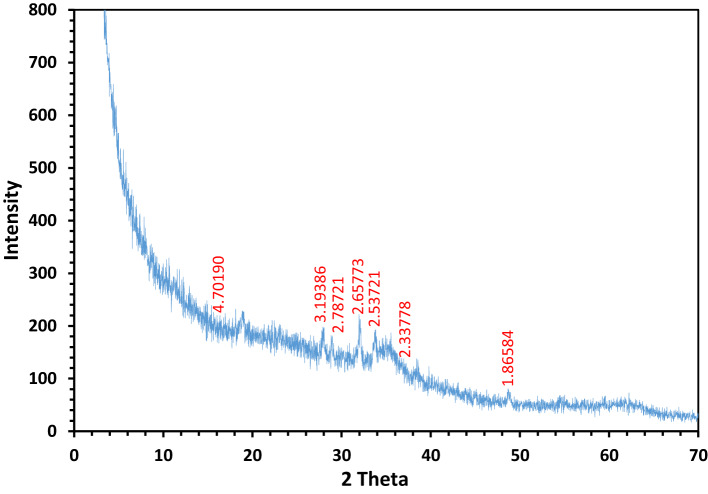
XRD analysis of NSM nanocomposite adsorbent.

The crystalline structure of the NSM was discussed using X-ray diffraction (XRD), as illustrated in Fig. 6. The XRD patterns of NSM were recorded over a 2*θ* range of 0° to 70°. The XRD pattern of the NSM exhibits a broadening of the peak around 2*θ* = 18°, indicating an increase in the amorphous nature of the NSM. This broadening is likely due to incorporating *N*,* N*’-methylenebisacrylamide polymer with Si, which enhances the amorphous characteristics by modifying intermolecular interactions within the final composite^33^. Diffraction peaks at 2*θ* = 27.71, 32.13, 35.57, and 62.01° confirm the presence of magnetite nanoparticles with a spinel structure, consistent with the JCPDS card No. 98-3969^45,46^. The Si-substitution in *N*,* N*’-methylenebisacrylamide polymer does not significantly affect the structure of the magnetite nanoparticles.

#### Magnetic properties of NSM nanocomposite

**Fig. 7:**
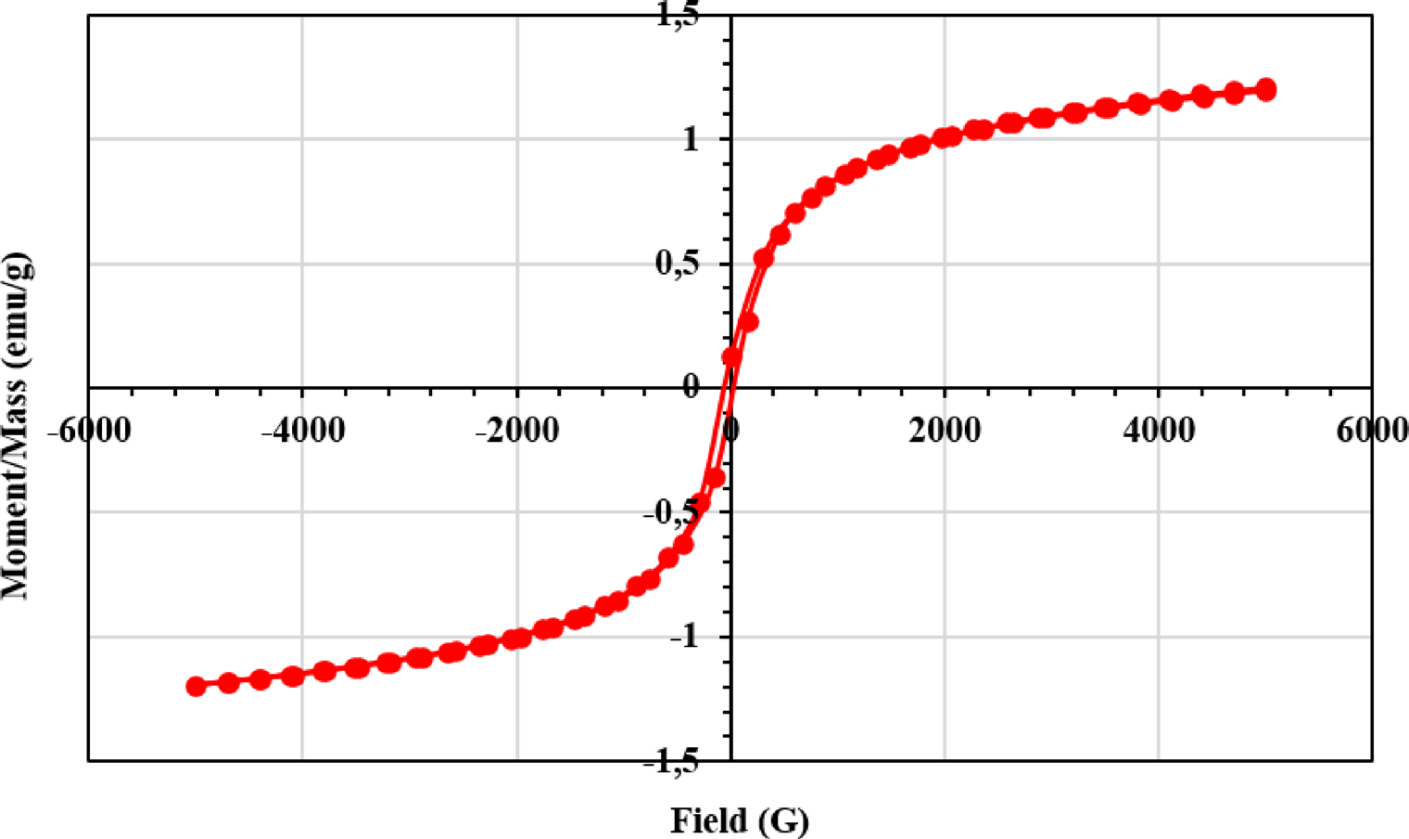
Magnetization curve for NSM nanocomposite adsorbent.

Several key magnetic properties must be considered to interpret vibrating sample magnetometer (VSM) data. The VSM measures the magnetization (M) of the sample as a function of the applied magnetic field (H), and the resulting data is presented as a hysteresis loop. This curve provides insight into how the studied sample responds to an external magnetic field, showing the extent of magnetization after removing the applied magnetic field^47^. Another important magnetic property is coercivity, which refers to the magnetic field required to decrease the magnetization to zero after the sample has been saturated^48^. This property is crucial in determining the magnetic softness or hardness of the material. Hard magnetic materials exhibit high coercivity and retain magnetization more effectively, whereas soft materials are characterized by low coercivity, indicating they can be easily demagnetized^49^. Saturation magnetization (Ms) represents the maximum magnetization that a material can attain when subjected to a strong magnetic field. Remanent magnetization or retentivity (Mr) refers to the residual magnetization remaining after the external magnetic field is removed, reflecting the material’s ability to retain magnetization. The shape of the hysteresis loop also provides insights into the material’s magnetic hardness, where a narrow loop indicates a soft magnetic material, while a wide loop suggests a hard magnetic material^50^. For the NSM nanocomposite, VSM experimental data were obtained with a magnetic field (H) ranging from − 5000 to 5000 Oe. As shown in Fig. 7, the saturation magnetization (Ms) is 1.2005 emu/g, coercivity is 42.814 Oe, and remanent magnetization (Mr) is 85.682 × 10^–3^ emu/g. The curve exhibits an S-shaped hysteresis loop with a wide width. The wide loop and relatively high coercivity suggest that the material is a hard magnetic adsorbent, facilitating its separation from the solution post-adsorption with an external magnet.

#### X-ray photoelectron spectroscopy (XPS) of NSM

**Fig. 8:**
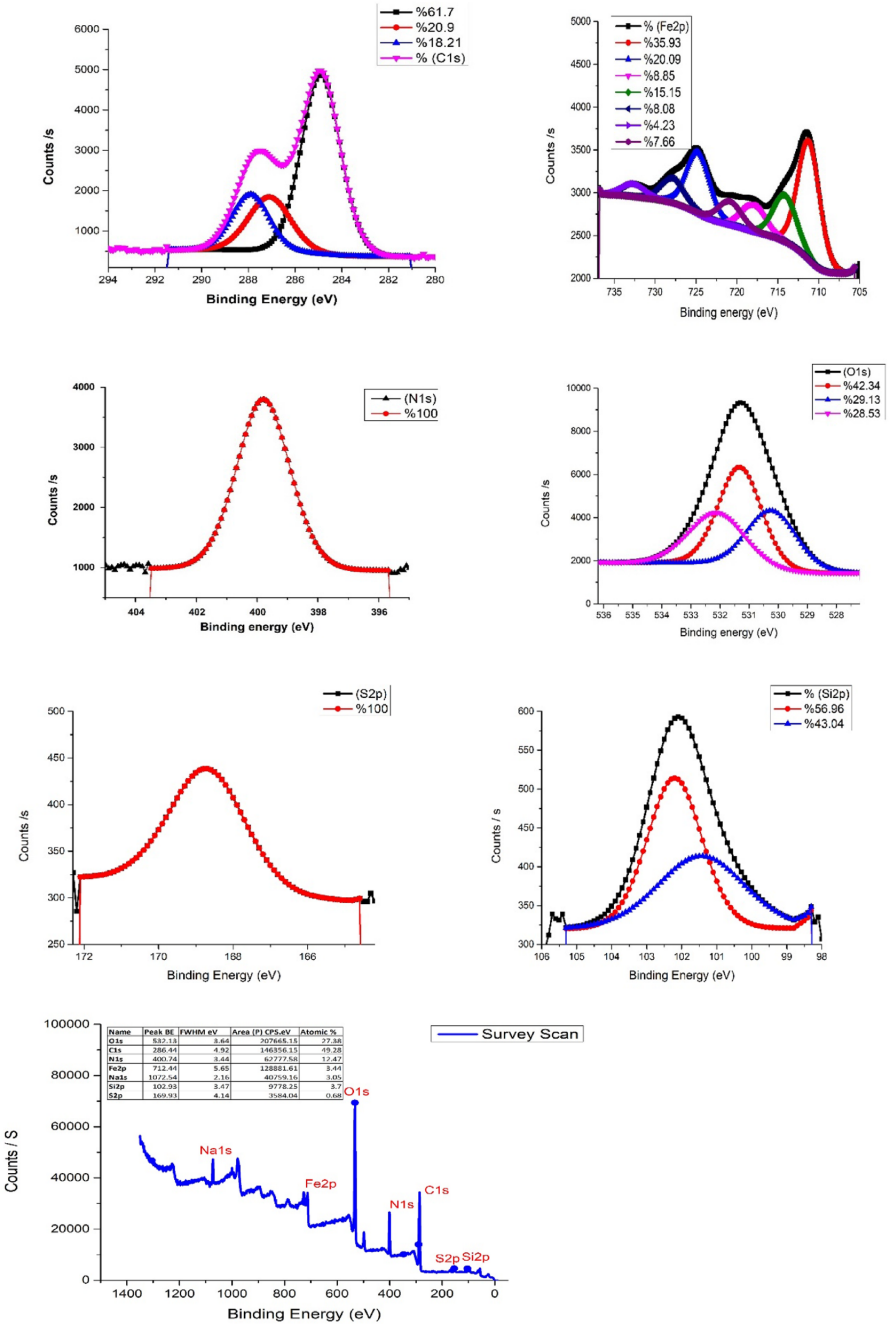
Overview of XPS spectrum of NSM nanocomposite adsorbent with 1 eV resolution.

The XPS is a surface-sensitive, quantitative analytical method employed to analyze materials’ elemental composition, chemical states, and electronic states. In an XPS spectrum, the X-axis denotes the binding energy (eV), which indicates the strength with which electrons are bound to atoms. The Y-axis signifies intensity, typically expressed in counts or arbitrary units corresponding to the number of electrons detected at a given binding energy^51^. Figure 8; Table 3 present the X-ray Photoelectron Spectrum of the NSM nanocomposite. The XPS spectrum confirms the presence of carbon at a binding energy of 286.44 eV, oxygen at 532.13 eV, and iron at 712.44 eV, corresponding to C 1s, O 1s, and Fe 2p orbitals, respectively, Fig. 8. The C 1s peak at 286.44 eV specifically represents carbon atoms in the carbonyl (C = O) functional group^52^. The O 1s peak exhibits two sub-peaks at 523.08 eV and 525.08 eV, corresponding to the C = O group^52,53^. The O 1s XPS spectrum of the magnetic Si-substituted poly(*N*,*N*’-methylenebisacrylamide) nanocomposite, shown in Fig. 8, reveals three peaks at binding energies of 523 eV, 525 eV, and 532.08 eV. The most prominent peak at 532.08 eV corresponds to lattice oxygen in Fe_3_O_4_^54^. The Fe 2p XPS spectrum exhibits peaks at binding energies of 704 eV, 721 eV, and 727.08 eV, corresponding to Fe 2p_3/2_, Fe 2p_1/2_, and Fe 2p_3/2_, respectively, confirming the presence of magnetite^52^. Si 2p and N 1s are represented by peaks at 102.93 eV and 400.74 eV binding energies, respectively.

**Table 3:** BET, BJH and XPS analysis of NSM nanocomposite adsorbent.

Name	Peak BE	FWHM eV	Area (*P*) CPS.eV	Atomic %
C1s	284.98	1.96	9488.67	61.70
	287.18	2.12	3086.19	20.09
	287.99	1.86	2795.43	18.21
Fe2p	711.3	3.14	4702.26	35.93
	724.84	3.37	2601.46	20.09
	718.08	3.37	1152.41	8.85
	714.21	3.37	1978.78	15.15
	728.01	3.37	1043.23	8.08
	732.62	3.37	544.88	4.23
	720.95	3.37	995.12	7.66
N1s	399.79	2.06	6316.9	100.00
S2p	168.74	2.4	334.77	100.00
O1s	531.39	1.76	8855.86	42.34
	530.32	2.01	6097.89	29.13
	532.18	2.29	5965.68	28.53
Si2p	102.28	1.83	385.12	56.96
	101.53	2.91	291.16	43.04

### Adsorption study

The batch equilibrium method was employed in this study to examine the adsorption of bivalent copper ions (Cu²⁺) onto the synthesized NSM adsorbent. Key experimental parameters, including contact time, solution pH, adsorbent dosage, and initial copper concentration, were carefully controlled throughout the study.

#### Effect of NSM adsorbent dosage

**Fig. 9:**
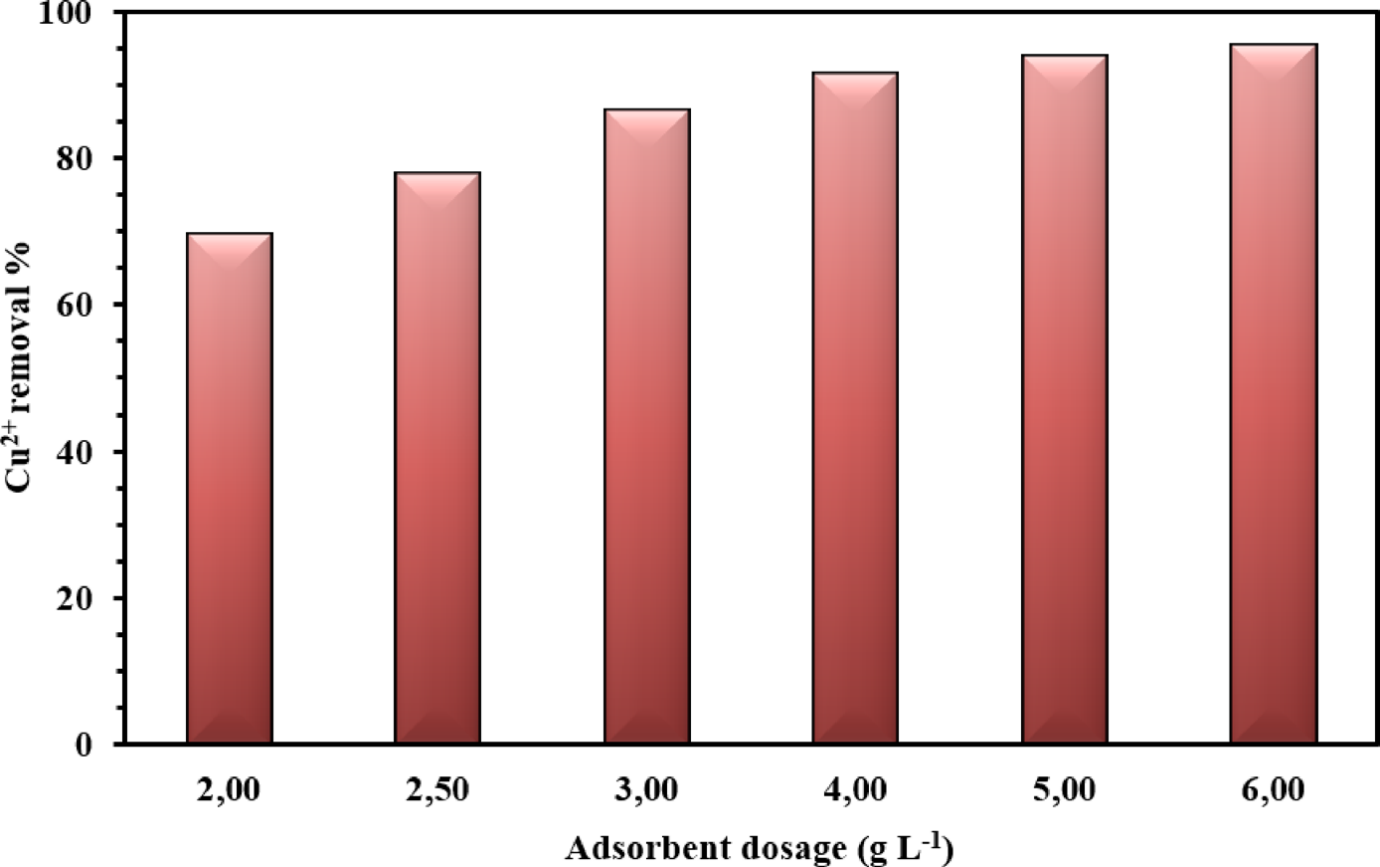
Effect of NSM nanocomposite doses (2.00–6.00 g L^–1^) of 75 mg L^–1^ Cu^2+^ ions concentration (The standard deviation is ± 1.8).

The work conditions were established to investigate the influence of NSM dosage on Cu^2+^ removal, Cu^2+^ ions starting concentration (50–150 mg L^–1^), NSM nanocomposite dosages (2.00–6.00 g L^–1^), work temperature (25 °C), contact time (60 min.) and the pH of the solution were adjusted to 5, and the results are presented in Fig. 9. Figure 9 illustrates that the removal percentage enhances with the amount of adsorbent across all initial Cu^2+^ ions concentration. Increasing the NSM nanocomposite adsorbent concentration from 2.0 to 6.0 g L^–1^, the removal % of Cu^2+^ ions adsorbed is calculated as 69.7 to 95.3%, respectively. This tendency is due to the increased availability of active binding sites as the adsorbent mass increases while the amount of Cu^2+^ ions remains constant.

#### Effect of contact time

**Fig. 10:**
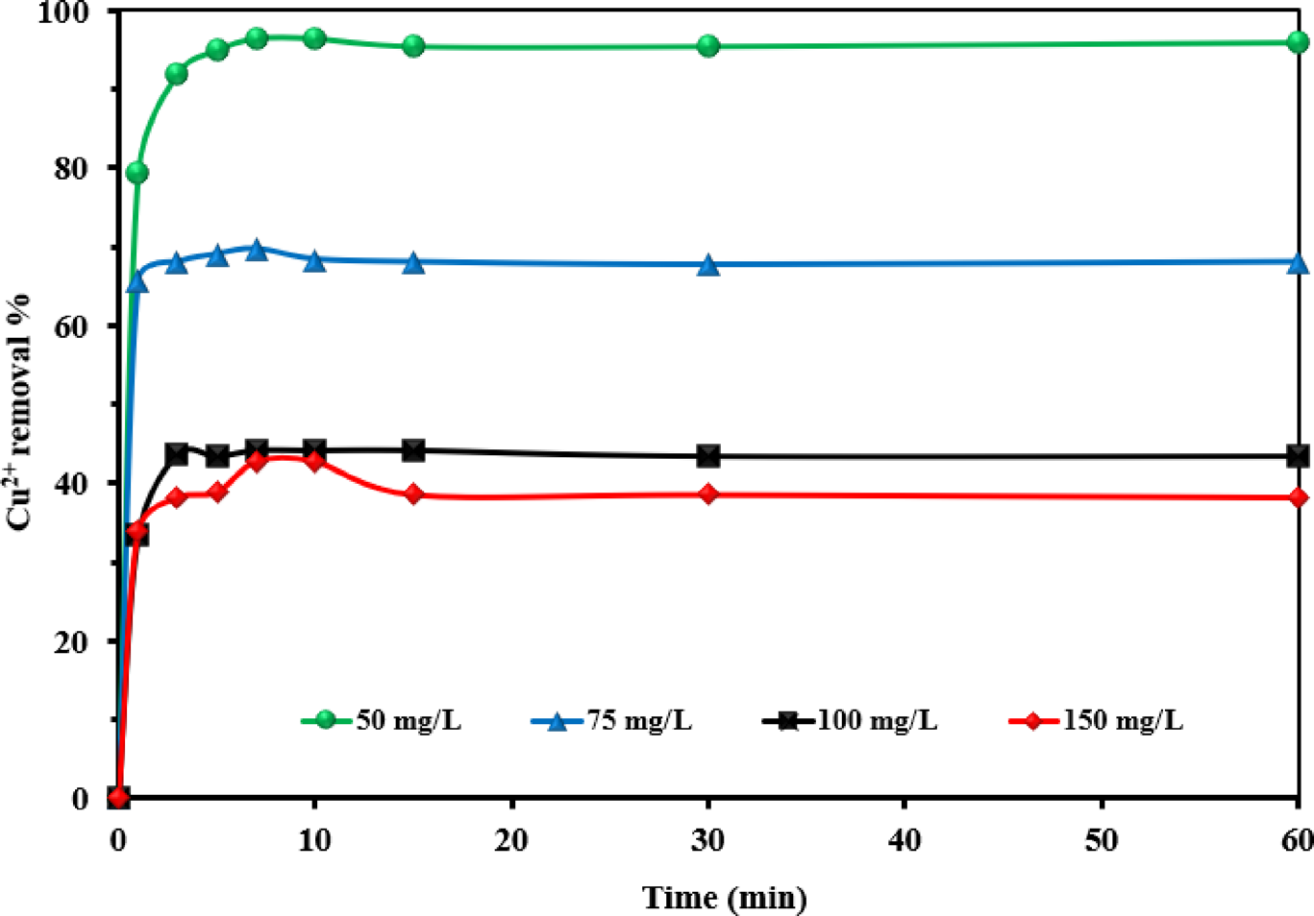
Removal of Cu^2+^ ions for 1 h using NSM nanocomposite adsorbent [Cu^2+^ (50–150 mg L^–1^), NSM nanocomposite dose (2.0 g L^–1^), Temp. (25 °C)].

The contact time with the NSM nanocomposite adsorbent is a critical parameter influencing the adsorption of Cu^2+^ ions. Studies were performed with initial Cu^2+^ ions concentrations between 50 and 150 mg L^–1^ to evaluate this effect, using an NSM nanocomposite dosage of 2 g L^–1^. Figure 10 illustrates the effect of contact time on the adsorption of bivalent copper ions onto the NSM nanocomposite adsorbent. The experimental data indicate rapid adsorption, with copper removal exceeding 40% of the initial concentration within the first few minutes. Adsorption reached equilibrium within approximately 10 min. with higher initial copper concentrations exhibiting lower removal percentages. The initial rapid adsorption rate can be attributed to the abundance of free binding sites on the adsorbent’s surface, which were initially vacant. As these sites became occupied over time, the number of available sites decreased, leading to a slower adsorption rate.

#### Effect of pH

**Fig. 11:**
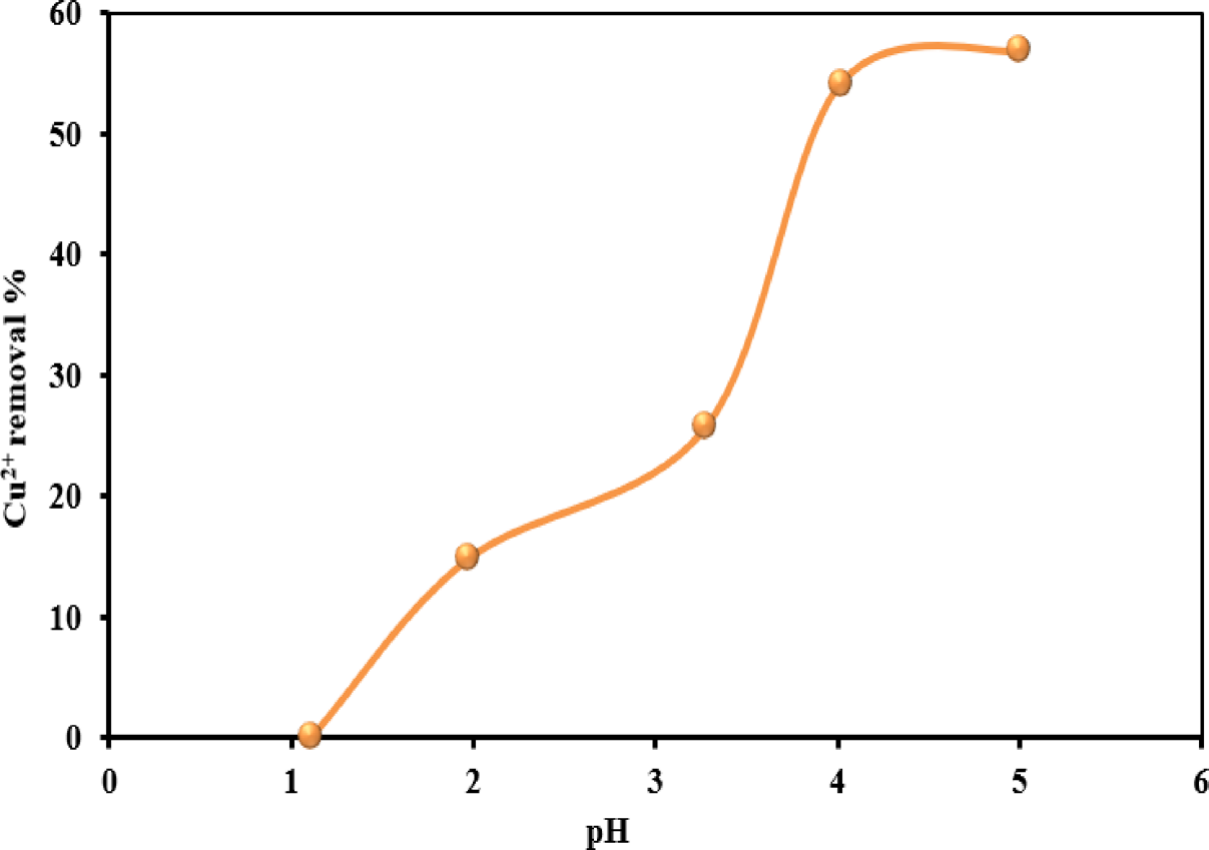
Adsorption of Cu^2+^ ions by NSM nanocomposite adsorbent as a function of pH on the removal percentage (The standard deviation is ± 2.3).

The initial pH of the solution is a crucial determinant in regulating and evaluating the adsorption process. It was observed that copper uptake by the NSM nanocomposite increased at relatively low pH values. It influences the selection of adsorbed copper species and affects the surface charges of the adsorbent^16^. In this study, initial pH values between 1 and 6 were applied, as pH levels above 6 could lead to the partial or complete precipitation of Cu^2+^ ions as Cu(OH)_2_, rendering the results unreliable^55^. Figure 11 shows the influence of pH on the adsorption of Cu^2+^ ions onto the NSM nanocomposite adsorbent. The experimental data indicate a sharp increase in copper adsorption at a pH of approximately 3.5. At lower pH values, adsorption efficacy increased gradually from pH 1 to 3.5. The relatively low adsorption observed at very low pH levels results from the elevated concentration of H^+^ ions, which compete with Cu^2+^ ions for active sites on the adsorbent surface. With increasing pH, the concentration of H^+^ ions decreased, reducing competition for active sites and enhancing adsorption.

### Adsorption kinetics

Kinetic models were utilized to evaluate the experimental outcomes and gain deeper insights into the adsorption process. The widely applied models in adsorption studies include the pseudo-first-order (PFOM), pseudo-second-order (PSOM), intraparticle diffusion (IPDM), and film diffusion (FDM) models. This study investigates the adsorption of copper ions onto the NSM nanocomposite by fitting the experimental data to PFOM, PSOM, IPDM, and FDM models (Tables 4 and 5). The PFOM is expressed by Eq. (4).

4 $$\:\text{log}\left({q}_{\text{e}}-{q}_{\text{e}}\right)=\text{log}\left({q}_{\text{e}}\right)-\frac{{k}_{1}}{2.303}t$$

Where *q*_t_ (mg g^− 1^) represents the amount of copper adsorbed onto the prepared nanocomposite at time (min), *q*_*e*_ (mg g^−1^) denotes the amount of copper adsorbed at equilibrium, and *k*_*1*_ (L min^−1^) is the equilibrium rate constant for the PFOM adsorption process.

**Fig. 12:**
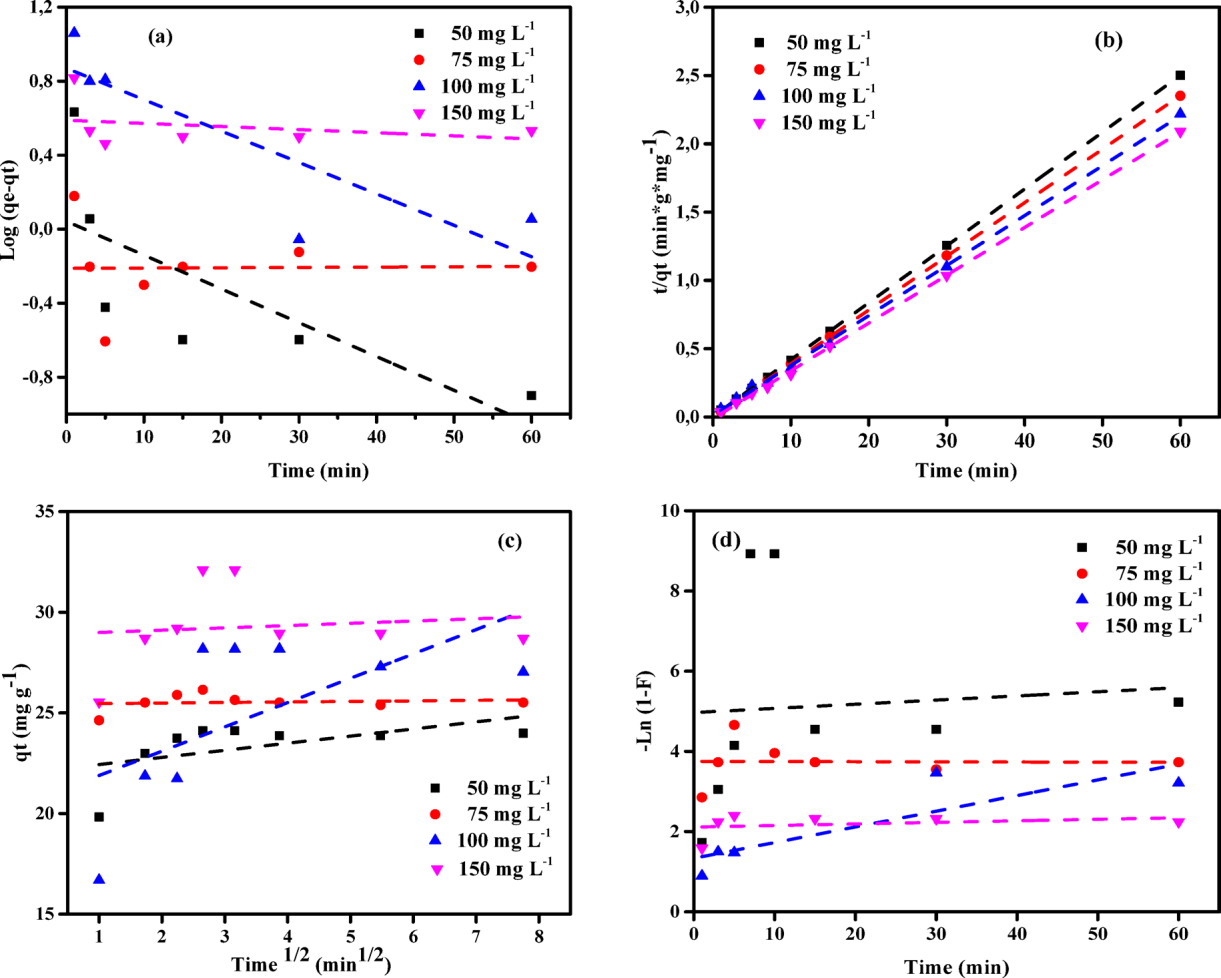
(**a**) PFOM, (**b**) PSOM, (**c**) IPDM, and (**d**) FDM models of adsorption of Cu^2+^ ions by NSM nanocomposite adsorbent (C_0_ = (50–150 mg L^–1^), Adsorbent dose = (2.00 g L^− 1^), Temp. = 25 °C).

Figure 12 demonstrates a linear correlation between log (*q*_e_ – *q*_t_) and *t*, from which key parameters such as *k*_1_ and *q*_e_ can be derived. The equilibrium rate constant *k*_*1*_ is derived from the slope of the graph, while *q*_e_ is obtained from the intercept. However, the experimental data indicate that the PFOM does not adequately characterize the adsorption process of copper ions onto the prepared magnetic adsorbents. This conclusion is supported by the relatively low correlation coefficient and the significant discrepancy between the calculated *q*_e_ values and the experimental *q*_e_ values.Pseudo-second order model (PSOM) is expressed in the Eq. (5):

5 $$\:\left(\frac{\text{t}}{{\text{q}}_{\text{t}}}\right)=\frac{1}{{\text{k}}_{2}{{\text{q}}_{\text{e}}}^{2}}+\frac{1}{{\text{q}}_{\text{e}}}\left(\text{t}\right)$$

where *q*_t_ (mg g^− 1^) represents the amount of copper adsorbed onto prepared nanocomposite at a given time *t* (min), *q*_e_ (mg g^− 1^) denotes the adsorption capacity at adsorption equilibrium, and *k*_2_ (g mg^− 1^ min^− 1^) represents the kinetic rate constant for the PSOM. The initial sorption rate, *h*, can be determined using Eq. (6):

6 $$\:h={k}_{2\:}{{q}_{\text{e}}}^{2}$$

Tables 4 and 5 demonstrate that the PFOM, IPDM, and FDM are inadequate for describing the adsorption of Cu^2+^ ions onto the NSM nanocomposite, whereas the PSOM offers a superior match. This conclusion is supported by the consistently high correlation coefficient (*R*^2^) values obtained for the PSOM and the excellent similarity between the experimental *q*_e_ and the predicted *q*_e_ using PSOM. Furthermore, the experimental adsorption values closely align with the calculated ones under this model. These observations strongly suggest that the PSOM is the most suitable model for accurately describing the adsorption system of Cu^2+^ ions onto the NSM nanoparticles.

**Table 4:** Results of the PFOM and PSOM kinetic models for the adsorption of Cu^2+^ ions by the NSM adsorbent [Initial concentration (50–150 Mg L^–1^), NSM nanocomposite doses (2.00–6.00 g L^− 1^), Temp. (25 °C)].

Parameter			PFOM			PSOM			
NSM conc. (g L^− 1^)	Cu^2+^ conc. (mg L^–1^)	q_e_ (exp.)	q_e_ (calc.)	k_1_ x 10^3^	*R* ^2^	q_e_ (calc.)	k_2_ x 10^3^	h	*R* ^2^
2.0	50	24.117	0.990	42.145	0.560	24.04	41.60	24.038	1.000
	75	26.145	26.145	0.461	0.000	25.45	39.30	25.445	1.000
	100	28.173	28.173	39.151	0.742	27.32	36.60	27.322	0.999
	150	32.102	32.102	3.915	0.088	28.57	35.00	28.571	1.000
2.5	50	19.394	5.460	2.533	0.087	19.31	51.80	19.305	1.000
	75	23.338	6.822	2.303	0.158	22.99	43.50	22.989	1.000
	100	24.456	1.876	1.612	0.000	23.87	41.90	23.866	1.000
	150	27.700	8.158	0.921	0.057	25.84	38.70	25.840	1.000
3.0	50	16.246	8.181	35.697	0.126	16.18	61.80	16.181	1.000
	75	21.635	3.165	9.673	0.011	20.96	47.70	20.964	1.000
	100	23.829	4.270	11.054	0.042	19.84	50.40	19.841	0.998
	150	25.859	6.966	24.182	0.034	25.25	39.60	25.253	1.000
4.0	50	12.185	35.694	9.903	0.005	12.06	82.90	12.063	1.000
	75	17.362	2.040	3.915	0.211	16.92	59.10	16.920	1.000
	100	19.322	1.169	136.338	0.931	18.94	52.80	18.939	1.000
	150	24.000	1.109	90.738	0.467	24.04	41.60	24.038	1.000
5.0	50	9.748	29.275	10.133	0.015	9.65	103.60	9.653	1.000
	75	14.092	63.665	58.496	0.160	13.77	72.60	13.774	1.000
	100	18.890	2.398	44.909	0.353	17.67	56.60	17.668	0.998
	150	22.430	36.191	37.539	0.367	22.03	45.40	22.026	0.999
6.0	50	9.798	8.890	17.733	0.483	9.75	102.60	9.747	1.000
	75	14.293	87.579	78.993	0.178	13.70	73.00	13.699	1.000
	100	19.445	19.838	7.139	0.002	18.90	52.90	18.904	1.000
	150	25.004	274.221	35.236	0.042	24.94	40.10	24.938	1.000

**Table 5:** IPDM and FDM kinetic model results of Cu^2+^ ions adsorption by NSM adsorbent [Initial concentration (50–150 Mg L^-1^), NSM nanocomposite doses (2.00–6.00 g L^− 1^), Temp. (25 °C)].

Parameter			IPDM			FDM		
NSM conc. (g L^− 1^)	Cu^2+^ conc. (mg L^–1^)	q_e_ (exp.)	K_dif_	C	*R* ^2^	K_FD_	C	*R* ^2^
2.0	50	24.117	0.35	22.08	0.286	0.0103	4.97	0.006
	75	26.145	0.03	25.44	0.018	0.0004	3.75	0.000
	100	28.173	1.21	20.68	0.384	0.0391	1.34	0.742
	150	32.102	0.11	28.88	0.014	0.0039	2.11	0.088
2.5	50	19.394	0.23	18.14	0.198	0.0026	1.49	0.087
	75	23.338	0.20	21.87	0.307	0.0002	1.45	0.158
	100	24.456	0.64	20.36	0.294	0.0017	3.83	0.000
	150	27.700	0.04	26.46	0.019	0.0009	1.45	0.057
3.0	50	16.246	0.12	15.57	0.260	0.0357	4.89	0.126
	75	21.635	0.12	20.47	0.142	0.0098	4.23	0.011
	100	23.829	0.08	20.15	0.014	0.0111	4.47	0.042
	150	25.859	0.12	24.73	0.091	0.0241	5.19	0.034
4.0	50	12.185	0.06	11.81	0.157	0.0064	4.36	0.022
	75	17.362	0.05	16.70	0.081	0.0038	3.57	0.021
	100	19.322	0.27	17.42	0.480	0.1364	3.09	0.931
	150	24.000	0.33	21.67	0.720	0.0908	3.26	0.467
5.0	50	9.748	0.01	9.65	0.056	0.0102	5.65	0.011
	75	14.092	0.02	13.99	0.080	0.0585	6.80	0.160
	100	18.890	0.33	16.00	0.423	0.0449	3.74	0.353
	150	22.430	0.13	21.81	0.065	0.0374	6.70	0.043
6.0	50	9.798	0.01	9.71	0.106	0.0178	4.47	0.483
	75	14.293	0.07	14.23	0.590	0.0791	0.28	0.282
	100	19.445	0.13	18.28	0.320	0.0009	6.53	
	150	25.004	0.14	24.26	0.191	0.0352	8.83	0.042

### Adsorption isotherms

**Fig. 13:**
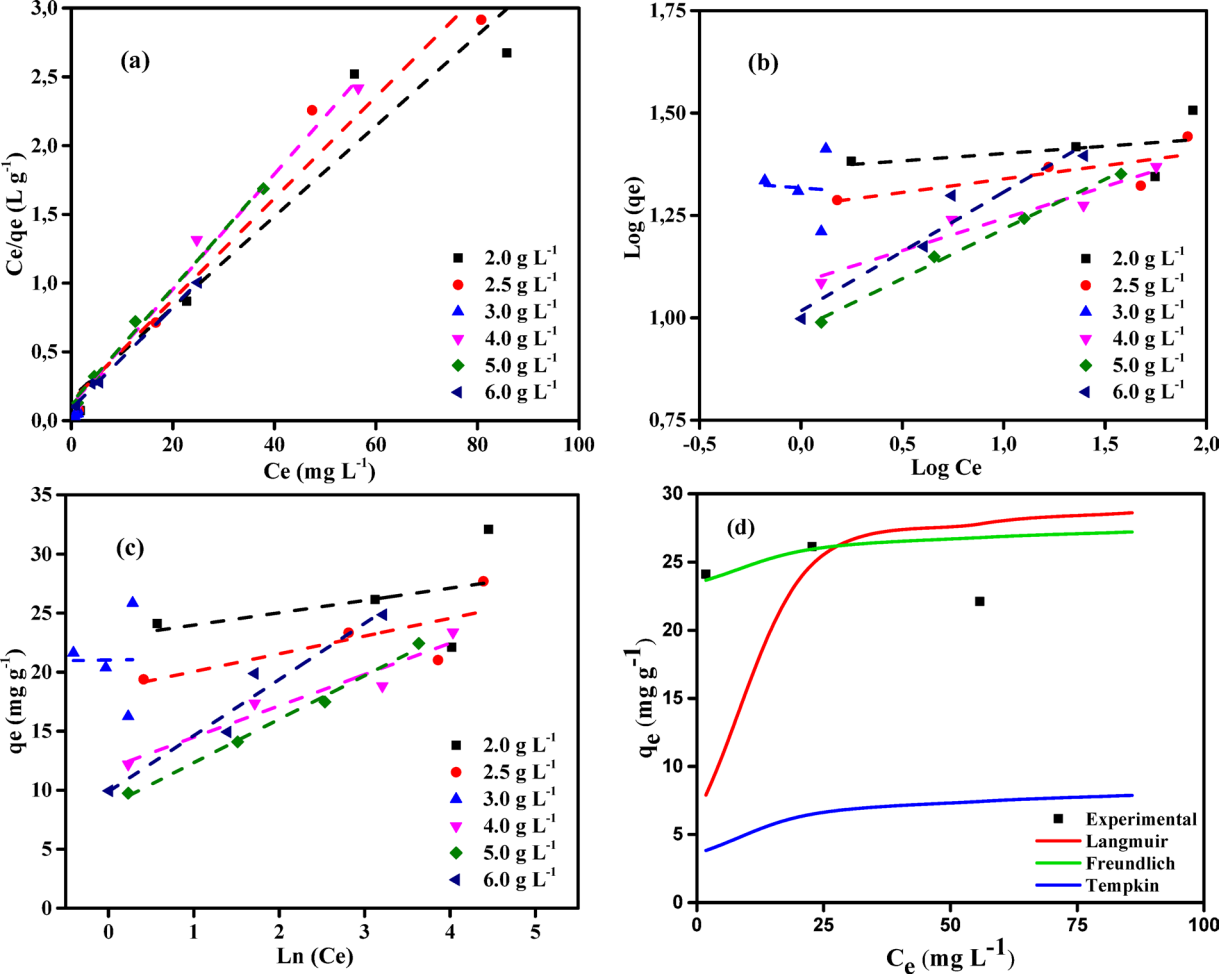
(**a**) LIM (**b**) FIM, and (**c**) TIM isotherm profiles for Cu^2+^ ions of initial concentration (50–150 mg L^− 1^) on NSM doses (2.00–6.00 g L^− 1^) at (25 °C), (**d**) Comparison of isotherm profiles at an adsorbent dosage of 2 g L^–1^ for the NSM nanocomposite adsorbent.

The main aim of studying isothermal models is to understand and explain the adsorption capacity of adsorbents with varying amounts of adsorbate under equilibrium conditions. Adsorption isotherms help to analyze the interaction between the adsorbate and the adsorbent. These isotherms are crucial for optimizing the use of adsorbents. The data derived from adsorption isotherms provide valuable insights into the interaction between the adsorbent and the adsorbate, and they also represent the adsorption affinity of the molecules involved^56^. In this study, we have examined the Langmuir (LIM), Freundlich (FIM), and Tempkin (TIM) models to better understand the adsorption process of copper ions on NSM nanoparticles (Fig. 13). These models were used to explore how Cu^2+^ ions interact with the adsorbent and to describe the adsorption isotherm, providing deeper insight into the adsorption behavior.

The LIM posits that a finite number of adsorption sites exists on the adsorbent’s surface. These active sites are uniform in size, energy, and shape, with each vacant site able to adsorb a single molecule of the adsorbate. This creates a homogeneous adsorbate monolayer on the adsorbent surface without any interactions between adsorbate molecules occupying different adsorption sites. The linear equation of the LIM can be expressed as^57^.

7 $$\:\frac{{\text{C}}_{\text{e}}}{{\text{q}}_{\text{e}}}=\frac{1}{{\text{K}}_{\text{L}}{\text{q}}_{\text{m}}}+\frac{1}{{\text{q}}_{\text{m}}}\times\:\text{C}\text{e}$$

Where *C*_e_ is the concentration of adsorbate in solution at equilibrium, *q*_m_ (mg g^–1^) represents the theoretical maximum adsorption capacity, *q*_e_ (mg g^–1^) indicates the equilibrium adsorption capacity, and *K*_L_ represents the Langmuir constant (L mg^–1^) associated with adsorption energy^58^.

The Freundlich isotherm is commonly employed to characterize the correlation between the quantity of adsorbate removed per unit mass of adsorbent at equilibrium and the concentration of the residual adsorbate in the solution.

8 $$\:\text{log}q\text{e}=\text{log}K\text{F}\:+\:\frac{1}{n}\:\text{log}C\text{e}$$

Where *n* represents a constant related to the relation between the adsorbate and adsorbent, *K*_F_ (mg g^–1^) denotes the Freundlich constant reflecting adsorption capacity, *q*_e_ (mg g^− 1^) represents the amount of Cu^2+^ ions removed per gram adsorbent at equilibrium, and *C*_e_ (mg L^–1^) signifies the equilibrium concentration of Cu^2+^ ions in the solution.

Table 6 presents experimental data and computed values about the isotherm models. The experimental data indicate that the LIM provides the best fit for describing Cu^2+^ ions adsorption in aqueous solution using the prepared NSM nanocomposite. The results suggest that the correlation coefficient for the LIM exceeds 0.919, whereas for the FIM, it is significantly lower at 0.150 for an adsorbent dosage of 2.00 g L^–1^. These results suggest that the adsorbent’s surface is homogeneous, with all adsorption sites exhibiting the same affinity for the adsorbate.

**Table 6:** Adsorption isotherm data for Cu^2+^ ions onto the NSM nanocomposite adsorbent [Cu^2+^ (50-150 mg L^–1^), adsorbent doses (2.00-6.00 g L^–1^), Temperature (25 °C)].

Isotherm model	Parameters	NSM doses (g L^–1^)					
		2.00	2.50	3.00	4.00	5.00	6.00
LIM	*Q*_*m*_ (mg g^− 1^)	30.30	27.10	20.08	23.87	23.92	27.10
	*K*_*L*_ x10^3^	0.20	0.26	10.25	0.36	0.33	0.43
	*R* ^*2*^	0.919	0.963	0.609	0.989	0.995	0.996
FIM	*1/n*	0.04	0.07	0.04	0.16	0.04	0.29
	*K*_*F*_ (mg^1 − 1/n^ L^1/n^ g^–1^)	23.20	18.77	20.76	76.97	23.20	10.39
	*R* ^*2*^	0.150	0.564	0.003	0.938	0.150	0.934
TIM	*A*_*T*_	21.97	12.33	185.29	4.46	2.35	2.08
	*B*_*T*_	1.04	1.50	0.11	2.66	3.68	4.75
	*R* ^*2*^	0.177	0.540	0.458	0.936	0.994	0.951

### Error function studies for best-fit isotherm model

To identify the optimal model for the adsorption of Cu^2+^ ions on NSM adsorbent, the correlation coefficients (*R*^2^) for the LIM, FIM, and TIM were evaluated against the experimental equilibrium data. An alternative approach to identifying the most appropriate isotherm model involves comparing error function values. To assess the error distribution between the predicted isotherm models and the experimental equilibrium data, commonly employed functions include average percent errors (*APE*), root mean square errors (*RMS*), sum of the errors squared (*ERRSQ*), the average relative error (*ARE*), Marquardt’s percent standard deviation (*MPSD*), Chi-square error (*X*^*2*^), sum of absolute errors (*EABS*), and hybrid error function (*HYBRID*)^59^. The terms of the error function unequivocally indicate that the LIM is the most suitable option (Table 7).

**Table 7:** Some error function values of the isotherm models are most appropriate for the experimental equilibrium data on the adsorption of Cu^2+^ ions on NSM nanocomposite.

Isotherm model	APE (%)	X^2^	Hybrid	ERRSQ	MPSD	ARE	EABS	RMS
LIM	0.353	3.786	16.460	73.472	1.842	0.353	42.858	1.767
FIM	3.442	353.095	1535.195	6852.404	17.788	3.412	413.896	17.062
TIM	2.463	183.999	799.997	3570.817	12.841	2.463	298.782	12.317

### Comparison with results reported in the literature

The literature review in Table 8 compares the efficacy of other adsorbents in removing Cu^2+^ ions with that of the NSM adsorbent, demonstrating that the NSM nanocomposite adsorbent is effective.

**Table 8:** Comparison of the Cu^2+^ ions *Q*_m_ of different adsorbents.

Adsorbent name	Maximum capacity (mg·g^− 1^)	Ref.
Tree fern	11.7	^60^
Orange peel	3.65	^61^
Watermelon rind	5.73	^62^
Banana peel	8.24	^62^
Sugarcane bagasse	9.48	^62^
Sawdust	1.79	^63^
Tea fungal biomass	4.64	^64^
Natural zeolite	141.12	^65^
Saccharomyces cerevisiae	1.90	^66^
Datura innoxia	7.20	^67^
NSM	30.30	This work

### RSM study

The chosen model was subjected to an ANOVA analysis to assess its relevance and identify the variables influencing the elimination percentage^23,24,30,31^. The experimental and anticipated elimination percentages and the ANOVA analysis outcomes are shown in Tables 9 and 10. The F-values show the significance of the variables and how they interact with the answer. A substantial impact of the factor or interaction on the answer is indicated by an F-value larger than 1. The Cu^2+^ removal % was most significantly impacted by the starting Cu^2+^ concentration, as Table 9 demonstrates. The model’s F-value of 155.93 (Table 10) emphasises its relevance.

Furthermore, factors are deemed significant if their p-values are less than 0.05. The relevance of the model is further supported by its p-value, which is less than 0.0001. The minor discrepancy between the adjusted *R*^2^ (0.9983) and projected *R*^2^ (0.9879), which is less than 0.2, further demonstrates the robustness of the model.

The following Eqs. (9, 10) for Cu^2+^ removal percentage were derived from the findings obtained:

9 $$\begin{aligned} {\text{Removal }}\% {\text{ for coded factors }} & = {\text{93}}.{\text{5}}0 + {\text{ 12}}.00{\text{A }} + {\text{ 2}}.{\text{43B }}{-}{\text{ 17}}.{\text{24C }} + {\text{ 1}}.0{\text{7AB }} \\ & + {\text{ 1}}0.{\text{68AC }}{-}{\text{ }}0.{\text{5}}0{\text{5}}0{\text{BC }}{-}{\text{ 6}}.{\text{12A}}^{{\text{2}}} {-}{\text{ 8}}.{\text{39B}}^{{\text{2}}} {-}{\text{ 8}}.{\text{66C}}^{{\text{2}}} \\ \end{aligned}$$

10 $$\begin{aligned} {\text{Removal }}\% {\text{ for actual factors }} & = {\text{ 76}}.{\text{64 }} + {\text{ 6}}.{\text{98 Dose }} + {\text{ 1}}.{\text{33 Time }}{-}{\text{ }}0.0{\text{68 Conc}}.{\text{ }} \\ & + {\text{ }}0.0{\text{37 Dose }} \times {\text{ Time }} + {\text{ }}0.{\text{1}}0{\text{7 Dose }} \times {\text{ Conc}}.{\text{ }} - {\text{ }}0.000{\text{7 Conc}}.{\text{ }} \\ & \times {\text{ Time }}{-}{\text{ 1}}.{\text{53 Dose}}^{{\text{2}}} {-}{\text{ }}0.0{\text{4}}0{\text{ Time}}^{{\text{2}}} - {\text{ }}0.00{\text{34 Conc}}.^{{\text{2}}} \\ \end{aligned}$$

**Table 9:** Experimental design for Cu^2+^ removal using NSM nanocomposite adsorbent.

Run	Factor 1 A: Dose (mg)	Factor 2 C: Time (min)	Factor 3 B: Conc. (mg/L)	Experimental removal %	Predicted removal %
1	4	30	150	61.98	61.13
2	4	15.5	100	93.5	93.50
3	6	15.5	150	83.35	84.16
4	6	15.5	50	97.48	97.28
5	2	1	100	65.68	65.64
6	2	30	100	67.71	68.36
7	4	15.5	100	93.5	93.50
8	4	15.5	100	93.5	93.50
9	4	1	150	57.44	57.28
10	2	15.5	50	95.46	94.65
11	4	15.5	100	93.5	93.50
12	6	30	100	94.45	94.49
13	4	15.5	100	93.5	93.50
14	4	30	50	96.47	96.63
15	2	15.5	150	38.6	38.80
16	6	1	100	88.14	87.49
17	4	1	50	89.91	90.76

**Table 10:** *ANOVA* analysis values determined by Box-Behnken design for influential variables in Cu^2+^ ion removal.

Source	Sum of squares	df	Mean square	F-value	*p*-value	
Model	4896.82	9	544.09	1023.71	< 0.0001	Significant
A-NSM dose	1151.28	1	1151.28	2166.14	< 0.0001	
B-Time	47.24	1	47.24	88.88	< 0.0001	
C-Cu dose	2378.78	1	2378.78	4475.68	< 0.0001	
AB	4.58	1	4.58	8.62	0.0219	
AC	456.46	1	456.46	858.84	< 0.0001	
BC	1.02	1	1.02	1.92	0.2085	
A^2^	157.51	1	157.51	296.36	< 0.0001	
B^2^	296.30	1	296.30	557.49	< 0.0001	
C^2^	315.86	1	315.86	594.30	< 0.0001	
Residual	3.72	7	0.5315			
Lack of fit	3.72	3	1.24			
Pure error	0.0000	4	0.0000			
Cor total	4900.54	16				
Std. Dev.	0.7290	R^2^	0.9992			
Mean	82.60	Adjusted R^2^	0.9983			
C.V. %	0.8826	Predicted R²	0.9879			
Adeq precision	104.5887					

**Fig. 14:**
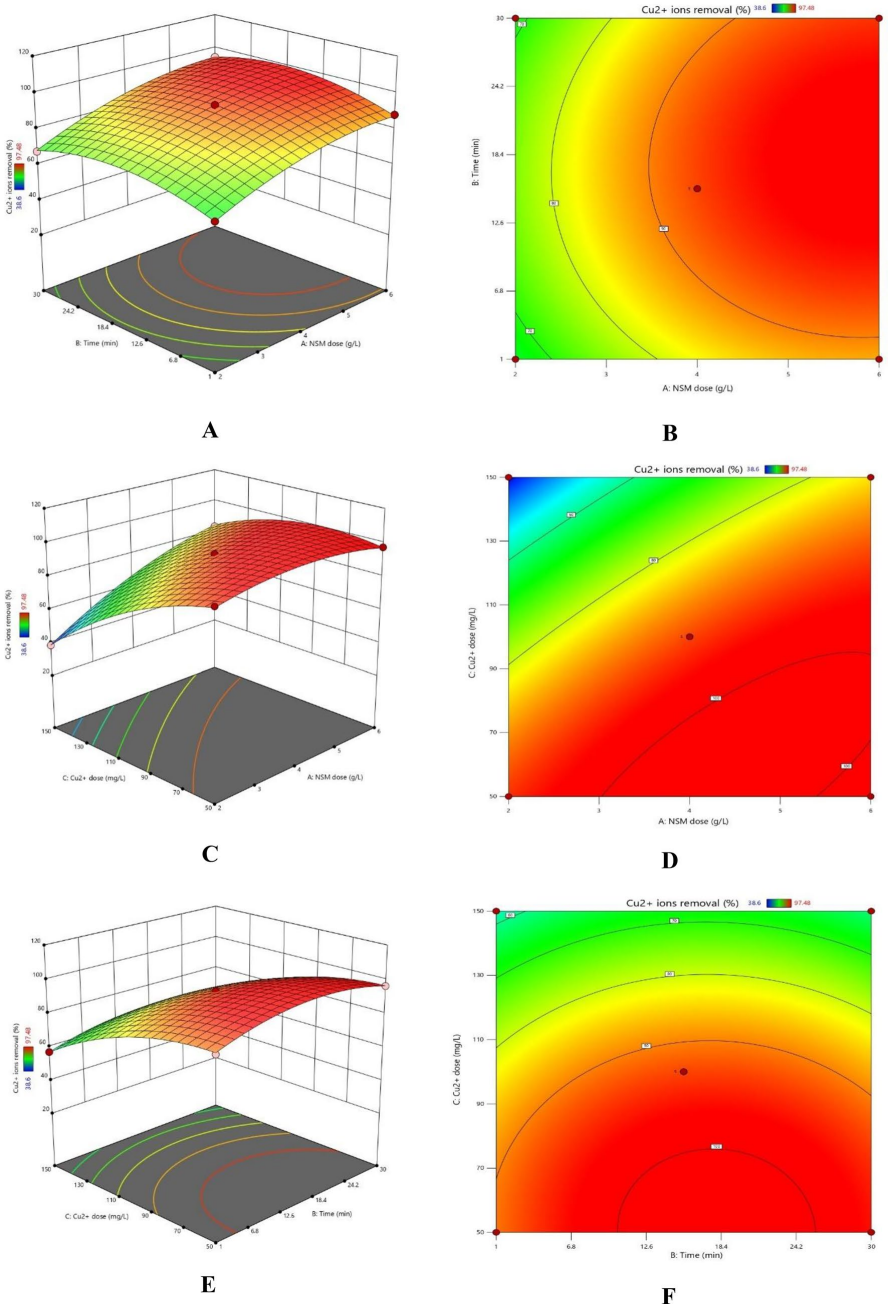
Combined effects of independent variables: (**A**, **B**) Adsorbent dose and time, (**C**, **D**) NSM Adsorbent dose and Cu^2+^ initial concentration, and (**E**, **F**) Cu^2+^ initial concentration and time.

The combined effects of reaction time, adsorbent dose, and beginning Cu^2+^ concentration on the percentage of Cu^2+^ ion removal are shown in Fig. 14. Low Cu^2+^ concentrations, large NSM adsorbent doses, and prolonged reaction durations all contribute to the best removal percentages^28,30,31^.

**Fig. 15:**
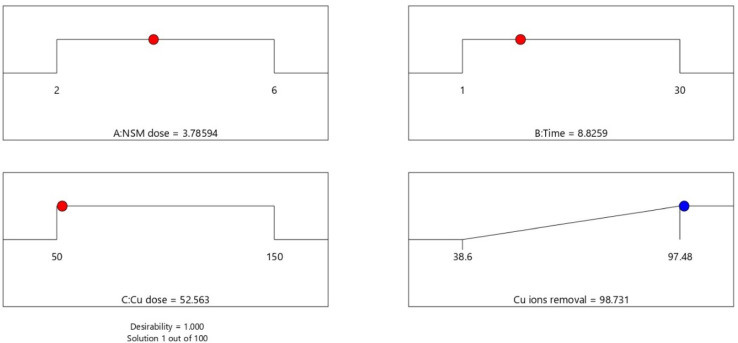
Optimization conditions through BBD settings.

The best operating settings to attain the maximum Cu^2+^ removal % were found statistically, as shown in Fig. 15.

### ANN modelling

**Fig. 16:**
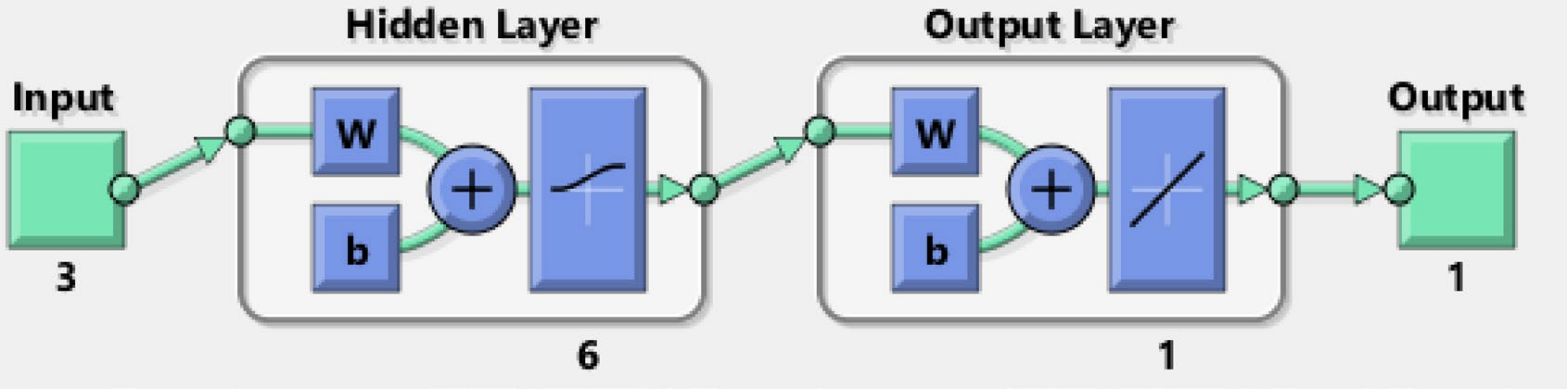
ANN architecture for H_2_ production.

**Fig. 17:**
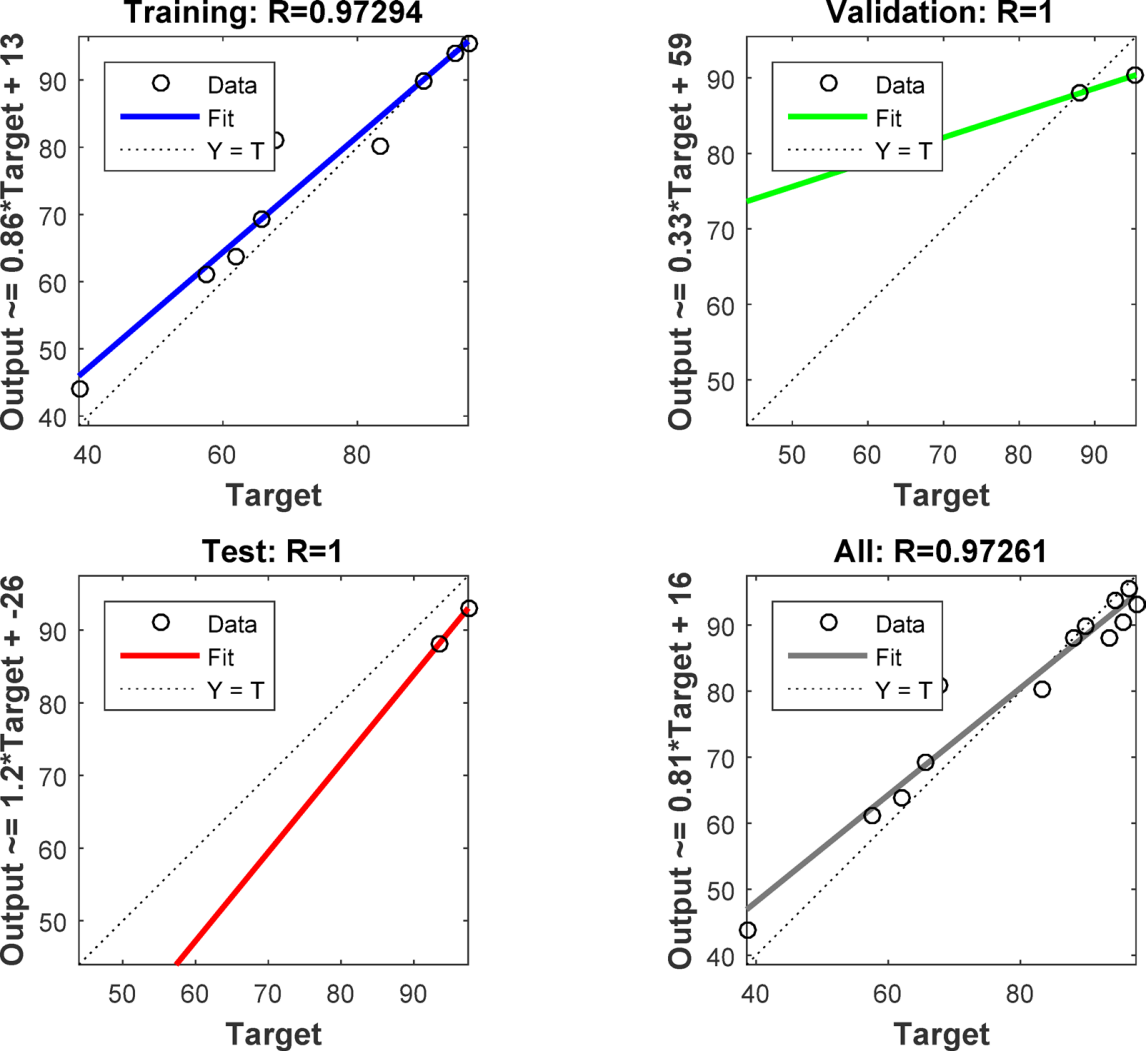
Training, validation, testing, and overall datasets for the LM algorithm.

**Fig. 18:**
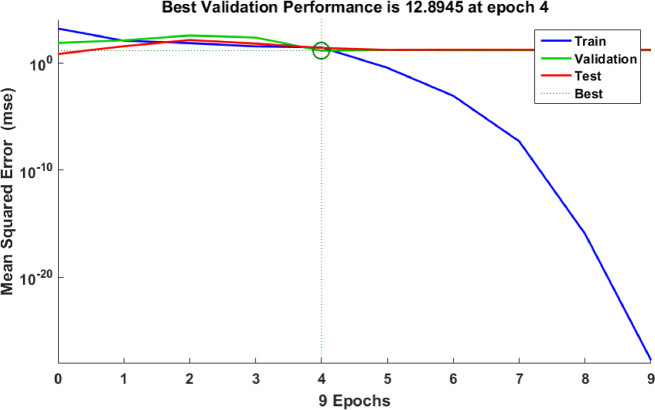
LM algorithm performance.

The Cu^+ 2^ adsorption sample data is 70:15:15 training, validation, and testing sample ratio. The optimal ANN model for Cu^2+^ adsorption using NSM was 3-6-1 (3- ILs, 6- HLs, and 1 OL) and trained by a backpropagation algorithm. Figure 16 illustrates the optimal ANN architecture. The. The regression plots of the ANN model in Fig. 17 possessed high *R*^2^ and low MSE error values. 0.97294, 1, 1, and 0.97261 were the *R*^2^ values for training, validation, testing, and overall. The MSE value was 1.91e-28. The NSM dosage (mg), Time (min), and initial concentration of Cu^2+^ (mg/L) were the inputs of the Cu^+ 2^ adsorption ANN approach. The Cu^2+^ removal was the output variable of the Cu^+ 2^ adsorption ANN approach. The Cu^2+^ adsorption best-fit ANN activation functions were Log-Sigmoid (log-sig) and Purelin for the hidden and output layers^68^. The results of the MSE error vs. the epoch number in Fig. 18 showed that Cu^+ 2^ adsorption’s best validation performance was at 4 epochs^69–71^.

### Desorption and regeneration studies of NSM

**Fig. 19:**
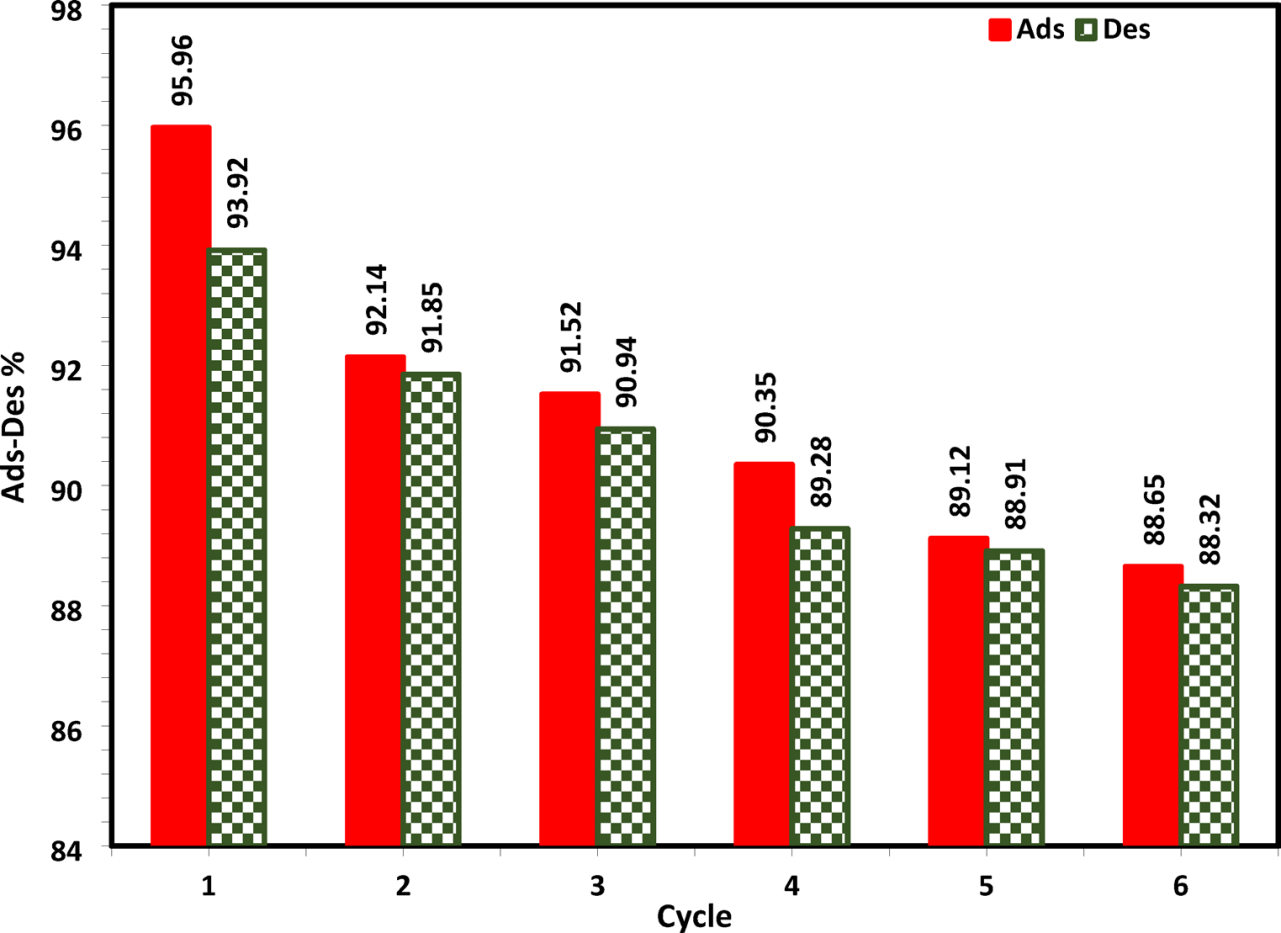
Cu^2+^ ions desorption (Des) % from NSM using 0.1 N HCl and 0.1 M NaOH, and Cu^2+^ ions adsorption (Ads) cycles by regenerated NSM by 0.1 N HCl and 0.1 M NaOH.

To examine the economic viability and reusability of NSM, desorption tests of Cu^2+^ ions from the loaded NSM were carried out using 0.1 N HCl and 0.1 M NaOH as eluted media^72^. Under these conditions, as the number of regeneration cycles increased, the desorption percentage dropped (Fig. 19). The regenerated NSM was used in six adsorption/desorption cycles in a row. The adsorption quantity demonstrated remained constant throughout the cycles, and after six generations, the adsorption capacity dropped by 7.31%, indicating that it may be employed as a sustainable method of removing Cu^2+^ ions (Fig. 19).

## Conclusion

In this study, the synthesis of a magnetic Si-substituted poly(*N*,* N*’-methylenebisacrylamide) (NSM) nanocomposite as an adsorbent was achieved via copolymerization. The synthesized NSM nanocomposite was comprehensively characterized using XRD, BET, XPS, SEM, FTIR, TGA and VSM. The material exhibited a magnetization value of 1.2005 emu/g. The NSM nanocomposite was successfully applied to eliminate copper ions (Cu^2+^) from contaminated water. The adsorption of Cu^2+^ ions was most efficient at a solution pH of 5, achieving a clearance rate of 56%. The NSM removal process has been studied in various settings, including initial Cu^2+^ ion concentration, pH, and temperature. Using an initial concentration of 50 mg L^–1^ of Cu^2+^ ions and a dose of 2.0 g L^–1^ NSM nanocomposite dose, the maximum percent clearance of Cu^2+^ ions was 96.47%. The *Q*_*m*_ of the NSM nanocomposite was 30.30 mg g^− 1^. Kinetic analysis demonstrated that the adsorption process adhered to the PSOM, while the LIM offered the most accurate representation of the experimental data, signifying monolayer adsorption on a homogeneous surface. Error function calculations indicate that the LIM is most suitable for describing adsorption. Furthermore, the nanocomposite demonstrated excellent reusability, as desorption of copper ions could be effectively achieved using sodium hydroxide, highlighting its economic value as a recyclable adsorbent for water treatment applications. In the end, RSM was used to optimize the parameters, and the results showed that utilizing 3.79 g of the NSM adsorbent and 52.65 ppm of Cu^2+^ solution in 8.83 min could get the highest degradation percentage (98.73%). For both the output and hidden layers, the best-fit ANN activation functions for Cu^2+^ adsorption were purelin and log-sigmoid (log-sig). The optimum validation performance of Cu^2+^ adsorption was seen at 4 epochs, according to the MSE error vs. epoch number findings.

Although the NSM nanocomposite demonstrated high adsorption efficiency for Cu²⁺ ions, future research should explore its performance in removing other heavy metals, organic pollutants, and mixed contaminants in complex real-water matrices. Investigations into surface modification or functionalization could enhance selectivity and adsorption capacity. Long-term stability, large-scale synthesis, and cost-effectiveness assessments are essential to facilitate industrial applications. Moreover, integrating NSM with continuous-flow water treatment systems and studying its regeneration cycles over extended use could provide insights for sustainable deployment. Finally, coupling experimental data with advanced predictive modeling and machine learning approaches may help optimize operational parameters for diverse environmental conditions.

## Data Availability

The corresponding author of the research can provide the datasets used in this study for review upon request.
